# Quorum sensing inhibitor-antibiotic conjugate suppresses virulence and eradicates intracellular *Staphylococcus aureus*

**DOI:** 10.1016/j.pscia.2026.100155

**Published:** 2026-09-28

**Authors:** Meiling Ma, Shihao Luo, Junshu Wang, Zhuang Nie, Shaonan An, Jianyu Wang, Xiangxiang He, Fei Da, Lin Yao

**Affiliations:** a Department of Medicinal Chemistry and Pharmaceutical Analysis, School of Pharmacy, Fourth Military Medical University, Xi'an, 710032, PR China; b Department of Pharmacy, Shaanxi University of Chinese Medicine, Xianyang, 712046, PR China; c Military Medical Innovation Center, Fourth Military Medical University, Xi'an, 710032, PR China; d Department of Radiation Medical Protection, School of Military Preventive Medicine, Fourth Military Medical University, Xi'an, 710032, PR China

## Abstract

*Staphylococcus aureus* (*S. aureus*) employs an array of virulence factors, predominantly governed by the accessory gene regulator (agr) quorum sensing (QS) system, to potentiate infection severity. Particularly challenging is the capacity of intracellular *S. aureus* to evade both antibiotic treatment and host immune clearance, often resulting in chronic or recurrent infections that complicate pathogen eradication *in vivo*. To overcome these limitations, we report the rational design of a QS inhibitor-antibiotic conjugate (QSI-A) for simultaneous attenuation of virulence and significant reduction of intracellular *S. aureu*s. This conjugate integrates synthetic autoinducing peptide (AIP) analogues-potent inhibitors of agr QS-with a rifalogue antibiotic via cathepsin-cleavable linkers, supporting intracellular activation within the proteolytic milieu of host cells. Using qRT-PCR, hemolysis assays, and LC-MS, we demonstrate that QSI-A markedly suppresses the expression of key agr components (*agrA* and *RNAIII*) and major virulence genes (e.g., *psmα*, *hla*, *pvl*), while substantially reducing *δ*-toxin production and markedly suppressing hemolytic and cytolytic activities. Mechanistic studies reveal that QSI-A efficiently enters infected host cells-either through direct uptake or co-internalization with bacteria, and undergoes intracellular processing that generates antibacterial activity, leading to effective killing of intracellular *S. aureus*. In a murine bacteremia model, QSI-A treatment significantly reduced bacterial loads, underscoring its therapeutic potential. These results establish QSI-A as a dual-mechanism anti-infective strategy that concurrently silences virulence expression and targets intracellular reservoirs. Moreover, this QSI-antibiotic conjugate platform holds broad applicability for treating other pathogens dependent on QS-regulated virulence.

## Graphical abstract

**Figure:**
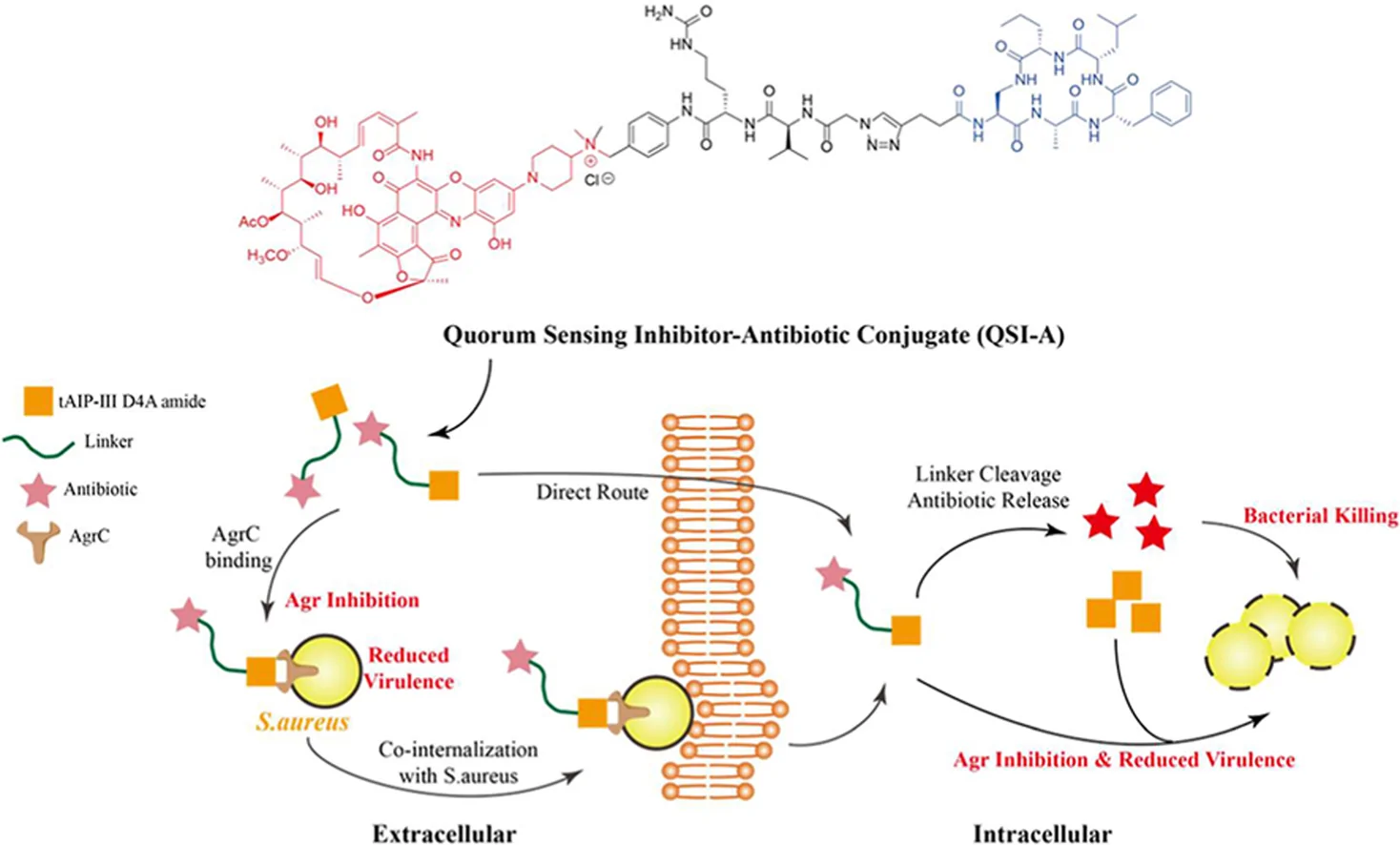

## 1 Introduction

*Staphylococcus aureus* (*S. aureus*) represents a formidable human pathogen, notorious for causing diverse infections ranging from skin and soft tissue infections to life-threatening bacteremia, endocarditis, and necrotizing pneumonia [1]. Compared to coagulase-negative *Staphylococci*, *S. aureus* is equipped with a diverse array of virulence factors, including a variety of toxins (such as Panton-Valentine leukocidin, adhesins, and immune evasion factors), which contribute to the exacerbation of infections [2]. In addition to its inherent antibiotic resistance [3], *S. aureus* has the capability to survive within host cells, thereby forming intracellular pathogens that can evade both antibiotic treatment and the host's immune response [4]. The intracellular *S. aureus* facilitate chronic, relapsing infections and complicates clinical eradication. Therefore, employing anti-virulence strategies and targeting intracellular bacteria represent promising approaches to combat infections caused by *S. aureus*.

In *S. aureus*, the accessory gene regulator (agr) quorum sensing (QS) system serves as a principal virulence regulator, playing a crucial role in invasive infections [5]. The agr system operates through a classic two-component signaling mechanism [6]. Specifically, *S. aureus* secretes autoinducing peptides (AIPs) that, upon reaching a critical threshold concentration, activate the transmembrane receptor AgrC. Once activated, AgrC phosphorylates the response regulator AgrA, which subsequently upregulates the P2 and P3 promoters as well as the expression of virulence factors known as phenol-soluble modulins (PSMs) [7,8]. The P3 locus is responsible for driving the expression of RNAIII, which serves as the primary effector of agr and activates various exotoxins such as hemolysins and enterotoxins [9].

Of note, *S. aureus* produces four distinct AIP types (I-IV), based on AIP-AgrC interactions, *S. aureus* divides into four agr groups (I-IV). AIPs are cyclic thiolactone peptides (7-9 amino acids) characterized by a central cysteine-linked ring and a variable tail dictating agr group specificity [6]. Cross-inhibition between groups occurs when exogenous AIPs block non-self AgrC activation, a trait explored for anti-virulence therapies [10].

Emerging studies have reported that commensal *staphylococci*-derived AIPs could competitively inhibit *S. aureus* AgrC, suppressing toxin production and colonization [[11], [12], [13]]. For instance, AIPs from *Staphylococcus simulans* reduces MRSA virulence and protects host skin from damage [14]. In addition, developing synthetic AIP derivatives that exploit this inhibitory mechanism represents a promising approach to disrupt QS and attenuate virulence without exerting bactericidal pressure. Recently, a series of synthetic AIP derivatives were designed and synthesized by Blackwell et al. to inhibit AgrC receptor. Among these peptides, the truncated AIP-III analogues-*t*AIP-III D4A amide, exhibits the most potent activities on inhibiting all four agr types by targeting conserved AgrC residues and reducing bacterial hemolysis [15].

Most currently used clinical antibiotics fail to eliminate intracellular bacteria due to their low accumulation efficiency within host cells [16]. The specific combination of AIP and AgrC suggests that AIP possesses the ability to target bacteria effectively, allowing it to label these microorganisms [17]. On one hand, it can antagonize the agr system; on the other hand, through its targeting capabilities, we suppose that it facilitates the delivery of antibacterial agents to the bacterial surface and enables these substances to be internalized into host cells with bacteria, thereby achieving a dual effect of counteracting toxicity and eliminating intracellular bacteria. In this study, we have developed a QS inhibitor-antibiotic conjugate (QSI-A) by covalently linking *t*AIP-III D4A amide, which specifically targets AgrC to block agr QS, to a rifamycin derivative (rifalogue) through a protease-cleavable linker. We evaluated its anti-virulence and intracellular antibacterial activities using *in vitro* infection models and a murine bacteremia model, with the aim of establishing a dual-mechanism strategy for combating *S. aureus* infections. Notably, QSI-A suppressed virulence by blocking agr QS and exerted intracellular bactericidal activity through intracellular activation within host cells.

## 2 Material and methods

All chemicals and solvents were of analytical grade, used as received, and sourced from commercial suppliers (Shanghai Adamas Reagent, Ltd.; Beijing InnoChem Science & Technology Co., Ltd.). The peptide, linker, and FITC-conjugated targeting peptide were custom-synthesized by Hangzhou Specialized Peptide Biotechnology Co., Ltd. (China). The rifalogue was purchased from Suzhou Medinoah Ltd. (China). Cell culture media, including Dulbecco's Modified Eagle Medium (DMEM) and fetal bovine serum (FBS) were procured from Gibco; Phosphate-buffered saline (PBS) from SRL. Tryptic Soy Broth (TSB) and Tryptic Soy Agar (TSA) from OXOID; and 1% Triton X-100 solution from Beyotime. Molecular biology reagents including the Cell Total RNA Isolation Kit (FOREGENE, China), 5×All-In-One RT MasterMix (abm, Canada), and BlasTaq™ 2×qPCR MasterMix (abm, Canada). Reverse-phase HPLC (analytical and preparative) was performed on a Kinetex 2.6 μm C18 100 Å (100 × 2.1 mm) column (Phenomenex) with water and acetonitrile (containing 0.1% TFA) as the mobile phase. Detailed instrument specifications and additional experimental procedures are provided in the Supporting Information.

### 2.1 Synthesis of QSI-A

QSI-A was prepared in three steps. First, the peptide *t*AIP-III D4A amide was conjugated to the linker via a Cu-catalyzed click reaction. Second, the resulting conjugate was treated with HCl/EA to convert the benzyl alcohol on the other side of the linker to benzyl chloride. Finally, the benzyl chloride intermediate was reacted with the rifalogue under basic conditions to afford the target conjugate QSI-A. The crude product was purified directly by reverse-phase preparative HPLC and analyzed by LC-MS and HPLC. Detailed synthetic methods are provided in the Supporting Information.

### 2.2 Determination of minimum inhibitory concentration (MIC)

The MIC of QSI-A against *S. aureus* was determined by the broth microdilution method. Two-fold serial dilutions of QSI-A in TSB were prepared in 96-well plates (quadruplicate). An exponential-phase culture of methicillin-resistant *S. aureus* USA300 LAC was adjusted to ∼1 × 10^6^ CFU/mL in TSB and added to each well. After shaking incubation at 37 °C for 18-24 h, bacterial growth was assessed by absorbance at 600 nm. The MIC was defined as the lowest QSI-A concentration inhibiting ≥90% of growth relative to the untreated control.

### 2.3 Cell counting Kit-8 (CCK-8) assay

Cell viability was assessed using the CCK-8 assay. Cells in exponential growth were seeded into 96-well plates at 2 × 10^4^ cells/well in 100 μL complete medium. After 24 h (37 °C, 5% CO_2_), the medium was replaced with serum-free medium containing QSI-A (0.5-10 μM) or vehicle control (0.1% DMSO). Following 24 h exposure, 10 μL CCK-8 reagent was added, and plates were incubated for 1-4 h at 37 °C in the dark. Absorbance was read at 450 nm. All treatments were in triplicate, and viability was expressed as a percentage of the vehicle control.

### 2.4 Lactate dehydrogenase (LDH) release

#### 2.4.1 Direct cytotoxic effect of QSI-A

The direct cytotoxicity of QSI-A on HaCaT cells was assessed via LDH release. Cells were seeded in 96-well plates at 5 × 10^4^ cells/well and allowed to adhere overnight. They were then treated with QSI-A (0.5-10 μmol/L, from a 2.4 mmol/L stock), along with vehicle control (0.4% DMSO), positive control (lysis solution), and blank (medium only). The CCK-8 assay measures mitochondrial dehydrogenase activity and reflects long-term cell viability; therefore, a 24h endpoint was chosen for that assay. In contrast, LDH release is an early marker of acute membrane damage. To detect this acute effect before potential enzyme dilution or degradation, we selected 1, 3, and 5 h time points. After incubation, 50 μL of supernatant was transferred to a new plate, mixed with an equal volume of LDH working solution, and incubated in the dark for 30 min. The reaction was stopped with 50 μL stop solution, and absorbance was read at 490 nm. Cytotoxicity was calculated as follows:

Cytotoxicity (%) = [(OD sample – OD blank) / (OD positive control – OD blank)] × 100

#### 2.4.2 Cytotoxicity of supernatant from QSI-A-treated bacteria

To evaluate cytotoxicity of bacterial metabolites modulated by QSI-A, culture supernatant from treated *S. aureus* LAC was collected and applied to HaCaT cells. Briefly, LAC (1 × 10^6^ CFU/mL) was incubated with QSI-A at various concentrations for 8 h at 37 °C with shaking (220 rpm). The culture was centrifuged (12,000 rpm, 10 min), and the supernatant was collected. HaCaT cells were incubated with this supernatant for 1 h, after which LDH activity was measured and cytotoxicity calculated as described in Section 2.4.1.

### 2.5 Hemolysis assay

#### 2.5.1 Direct hemolytic toxicity of QSI-A

Human erythrocytes were isolated from heparinized whole blood by centrifugation (500×g, 5 min) and washed three times with PBS (pH 7.4) until the supernatant was clear. The washed erythrocytes were resuspended in PBS to an 8% (v/v) suspension. For the assay, an 8% erythrocyte suspension was mixed 1:1 with QSI-A, resulting in a final erythrocyte concentration of 4% and incubated at 37 °C for 1 h. Negative and positive controls were PBS and 1% Triton X-100, respectively. After incubation, the mixtures were centrifuged (300×g, 5 min, 4 °C), and the absorbance of the supernatant was measured at 405 nm. Hemolysis (%) = [(OD sample – OD negative control)/(OD positive control – OD negative control)]×100.

#### 2.5.2 Hemolytic activity of supernatant from QSI-A-treated bacteria

*S. aureus* LAC (1 × 10^6^ CFU/mL) was incubated with or without QSI-A for 8 h at 37 °C with shaking (220 rpm). The culture supernatant was collected by centrifugation (12,000 rpm, 10 min, 4 °C). Then, 200 μL of the supernatant was mixed with 200 μL of 8% erythrocyte suspension and incubated at 37 °C for 1 h. Centrifugation, absorbance measurement (405 nm), and hemolysis calculation were performed as in Section 2.5.1.

### 2.6 Cathepsin release assay for QSI-A

QSI-A (200 μg/mL) was incubated in cathepsin buffer (pH 5.0) with or without cathepsin B (10 μg/mL) at 37 °C for 1 h. The reaction was terminated by adding nine volumes of TSB. Serial dilutions of the mixture were prepared in a 96-well plate, and USA300 LAC (2 × 10^3^ CFU/mL) was added. After overnight incubation at 37 °C with shaking, bacterial growth was measured by absorbance at 630 nm.

### 2.7 Plasma stability assay

Rat plasma was obtained from Sprague-Dawley rats (male, 8-10 weeks old, 200-250 g) housed at the Laboratory Animal Center of the Fourth Military Medical University. Blood was collected via cardiac puncture under anesthesia, and plasma was separated by centrifugation (3000 rpm, 5 min). All procedures were approved by the ethics committee of the Fourth Military Medical University (Approval no. KY20212479-C-1). QSI-A was incubated at 37 °C in 50% rat plasma/50% TSB. Aliquots were collected at 0, 3, and 6 h, and the remaining antibacterial activity was determined by MIC against *S. aureus* LAC as described in Section 2.2. The change in MIC over time indicated plasma stability.

### 2.8 RNA extraction and quantitative real-time PCR

#### 2.8.1 RNA extraction from bacteria

Bacteria were grown to mid-log phase (OD_600_ = 0.5-0.6), adjusted to 0.5 McFarland, and mixed 1:1 with QSI-A (final 1-0.001 μmol/L) or PBS (control) in a 96-well plate. After 5 h at 37 °C, cells were centrifuged (12,000 × g, 2 min, 4 °C). The pellet was lysed with lysostaphin (0.1 mg/mL) and lysozyme (15 mg/mL) for 15-30 min at room temperature, then RNA was purified using a commercial kit (FOREGENE).

#### 2.8.2 Reverse transcription and quantitative PCR (*RT*-qPCR)

cDNA was synthesized from 2 μg total RNA using the 5× All-In-One RT MasterMix. qPCR was performed on a QuantStudio 7 Pro system with BlasTaq™ 2× qPCR MasterMix in a 20 μL reaction containing 2 μL cDNA. Cycling: 95 °C for 3 min, then 40 cycles of 95 °C for 15 s and 60 °C for 1 min. Primers are listed in Table S2. Relative expression was calculated using the 2^−ΔΔCt^ method with gyrB as the endogenous control.

### 2.9 Detection of δ-toxin in bacterial culture supernatants

*δ*-Toxin levels were measured by RP-HPLC/ESI-MS on an ExionLC-X500R QTOF system. Samples (5 μL) were injected onto a Kinetex C18 column (2.6 μm, 100 Å, 100 × 2.1 mm) and eluted with a 0.1% formic acid water/acetonitrile gradient at 0.4 mL/min. Relative *δ*-toxin production was determined from the extracted ion chromatogram of multiply-charged *m*/*z* values using SCIEX OS software.

### 2.10 Biofilm formation assay

To evaluate the effect of QSI-A on biofilm formation, an *in vitro* crystal violet staining assay was performed. *S. aureus* LAC was cultured overnight in TSB at 37 °C with shaking, then diluted 1:100 in fresh TSB containing 5% glucose. Aliquots (100 μL) of the bacterial suspension were added to 96-well polystyrene plates and incubated with QSI-A at concentrations corresponding to 1/8 MIC (0.3125 μmol/L), 1/4 MIC (0.625 μmol/L), and 1/2 MIC (1.25 μmol/L) of QSI-A against LAC (MIC = 2.5 μmol/L). Untreated bacteria served as the negative control. Plates were incubated statically at 37 °C for 24 h to allow biofilm formation. After incubation, the culture medium was gently removed, and the wells were washed three times with PBS to remove non-adherent bacteria. The adherent biofilms were fixed with methanol for 15 min, air-dried, and then stained with 0.5% (w/v) crystal violet solution for 15 min at room temperature. Excess stain was removed by washing three times with distilled water. The crystal violet dye bound to the biofilm was solubilized with 200 μL of 33% (v/v) glacial acetic acid per well, and the absorbance was measured at 570 nm using a microplate reader. All experiments were performed in triplicate with at least three independent biological replicates.

### 2.11 Intracellular *S. aureus* killing assay

The intracellular activity of QSI-A was assessed in macrophages and non-phagocytic cells. Logarithmic-phase *S. aureus* (1 × 10^6^ CFU/mL) was added to cell monolayers (200 μL per well in 96-well plates, containing 2 × 10^5^ CFU per well) for 1 h to establish infection. For RAW264.7 macrophages (seeded at 5 × 10^4^ cells per well), this corresponds to an MOI of 4; for A549, HaCaT, and MG63 cells (seeded at 2 × 10^4^ cells per well), the same inoculum corresponds to an MOI of 10. Extracellular bacteria were removed by washing, followed by gentamicin (0.5 mg/mL, 30 min). Cells were then washed and treated with QSI-A, vancomycin, or medium alone for 4 h. After lysis with 1% Triton X-100, lysates were diluted, plated on TSA, and incubated overnight. Intracellular bacteria were quantified by CFU counting.

### 2.12 Intracellular activation assay for QSI-A

To assess whether intracellular processing of QSI-A generates antibacterial activity, RAW264.7 macrophages were seeded in 96-well plates at 5 × 10^4^ cells per well and allowed to adhere overnight. The cells were then incubated with QSI-A at concentrations of 2 μmol/L for 1 h at 37 °C. After incubation, the culture medium was removed, and the cells were gently washed twice with PBS to eliminate any extracellular QSI-A. The cells were then lysed with 0.1% Triton X-100 (200 μL per well) to release intracellular contents. The resulting lysates were collected, serially diluted, and mixed with an equal volume of freshly prepared *S. aureus* LAC suspension (final bacterial concentration adjusted to ∼1 × 10^6^ CFU/mL). A control group without QSI-A treatment was included in parallel. After co-incubation at 37 °C for 18 h, bacterial growth was assessed by visual inspection of turbidity. The MIC was defined as the lowest concentration of the cell lysate that completely inhibited visible bacterial growth.

### 2.13 Intercellular dissemination assay

C57BL/6J mice were infected intraperitoneally with *S. aureus* (1 × 10^8^ CFU). After 24 h, peritoneal lavage was performed with ice-cold PBS. Lavage fluid was centrifuged (1000×g, 5 min, 4 °C), and the cell pellet was treated with lysostaphin (50 μg/mL, 30 min) to eliminate all extracellular and surface-adherent bacteria, ensuring that only bona fide intracellular bacteria within macrophages were carried forward. After two washes, peritoneal macrophages were co-cultured with MG63 osteosarcoma cells (1:1 ratio) in 96-well plates. After 24 h, the non-adherent macrophages were removed by gentle washing, and only the adherent MG63 cells were lysed with 0.1% Triton X-100. Because no extracellular bacteria were present and macrophages were not lysed, the recovered CFU exclusively represent bacteria that had disseminated from macrophages into MG63 cells. The co-culture was treated with QSI-A, rifalogue, vancomycin, or PBS (control). After 24 h, MG63 cells were lysed with 0.1% Triton X-100, and intracellular bacteria were quantified by colony-forming unit (CFU) enumeration on TSA.

### 2.14 Mouse bacteremia infection model

Female BALB/c mice (6 weeks old, 18-20 g) were obtained from Charles River and housed under specific pathogen-free conditions at laboratory animal center of the Fourth Military Medical University under a 12h light/dark cycle at 22 ± 2°C and 50% ± 10% humidity, with ad libitum access to food and water. After a 7 day acclimatization period, *S. aureus* USA300 LAC was streaked on TSA plates, and a single colony was inoculated into TSB and grown at 37°C with shaking at 200 rpm to the logarithmic phase. Bacteria were harvested by centrifugation, washed, and resuspended in PBS, and the inoculum was adjusted to 1 × 106 CFU/100 μL and verified by serial dilution and plating. Mice were infected by tail vein injection with 100 μL of the bacterial suspension. Twenty-four hours post-infection, mice were randomly assigned to five groups (n = 3 per group) and received a single intraperitoneal dose of QSI-A (1.0 mg/kg), rifalogue (0.5 mg/kg), vancomycin (110 mg/kg), a physical mixture of *t*AIP-III D4A amide and rifalogue (at a molar ratio equivalent to that in QSI-A), or PBS. The administration volume was 10 mL/kg. At 72 h after dosing, mice were anesthetized with 2% isoflurane and humanely euthanized. Blood, liver, kidney and lung tissues were collected. Tissues were homogenized in PBS, serially diluted, and plated on TSA. Plates were incubated at 37°C for 12 h, and colonies were counted. The study is reported following the ARRIVE guidelines 2.0 where applicable and all animal experiments were performed in accordance with the national regulations on animal studies and were approved by the Medical Ethics Committee of the Fourth Military Medical University (Approval no. KY20212479-C-1).

### 2.15 Flow cytometry binding assay

#### 2.15.1 Bacterial binding assay

Mid-log phase *S. aureus* (or *E. coli* ATCC 25922) were washed, resuspended in PBS, and incubated with 5 μmol/L FITC-conjugated targeting peptide for 1 h at 37 °C in the dark. Bacteria were then washed twice, resuspended in PBS, and analyzed. An unstained control (PBS only) was included.

#### 2.15.2 Cellular binding assay in RAW264.7 macrophages

RAW264.7 murine macrophages were harvested, washed, and resuspended in PBS. Cells were incubated with the FITC-conjugated targeting peptide at final concentrations of 0.5 or 1 μmol/L for 1 h at 37 °C in the dark. After incubation, cells were washed twice with PBS and resuspended in PBS for analysis. Cells treated with PBS alone served as the unstained control.

#### 2.15.3 Flow cytometry analysis

All samples were analyzed immediately using a Beckman Coulter CytoFLEX flow cytometer. Green fluorescence was detected with excitation at 488 nm and emission collected at 533 ± 30 nm. Peptide binding was quantified as the mean fluorescence intensity (MFI), and data were processed using CytExpert software (Beckman Coulter).

### 2.16 Confocal live-cell imaging

Samples were prepared as described for flow cytometry. Live-cell images were acquired on a ZIRCON SC3000 confocal microscope with a climate chamber at 37 °C. FITC was excited at 488 nm, and emission was collected at 500-550 nm, with simultaneous bright-field imaging. Scale bars = 20 μm.

### 2.17 Statistical analysis

All experiments were performed with at least three independent replicates (N ≥ 3), unless otherwise specified in the figure captions or methods. Data are presented as mean ± SD. Statistical analyses were performed using GraphPad Prism version 9.5. For comparisons between two independent groups, an unpaired two-tailed Student’s *t*-test was used. For comparisons among multiple groups against a single control, one-way ANOVA followed by Dunnett’s multiple-comparisons test was used. A *P* value < 0.05 was considered statistically significant.

## 3 Results

### 3.1 Rifalogue demonstrates superior antibacterial activity against intracellular *S. aureus*

Intracellular *S. aureus* is widely reported to exhibit enhanced resistance to conventional antibiotics. To evaluate the efficacy of antibiotics against intracellular bacteria, we determined the MICs of six clinical antibiotics-vancomycin, tetracycline, imipenem, cefoperazone, rifampicin, and rifalogue, as summarized in Table S1. Subsequently, murine RAW264.7 macrophages infected with MRSA USA300 LAC were treated with each antibiotic at a concentration of 32×MIC. CFU assays revealed that rifalogue achieved the greatest reduction in intracellular bacterial burden among all tested agents (Fig. 1A). Furthermore, we assessed the MIC of rifalogue against *S. aureus* in the presence of varying concentrations of the AIP analog *t*AIP-III D4A amide. Notably, the MIC decreased two-to eight-fold as the *t*AIP-III D4A amide concentration increased from 0.5 to 5 μmol/L (Fig. 1B).

**Fig. 1:**
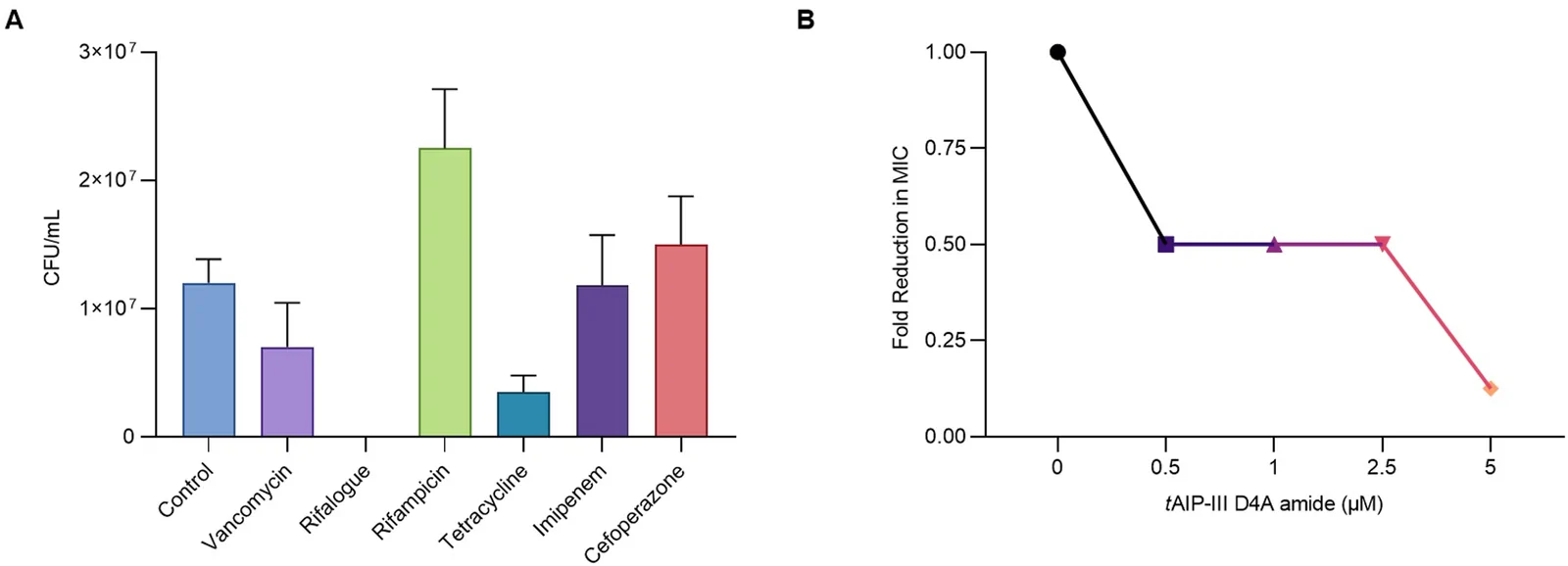
Rifalogue demonstrates superior antibacterial activity against intracellular *S. aureus*. **(A)** Murine monocyte-macrophage leukemia cells (RAW264.7) were infected with *S. aureus* LAC at a multiplicity of infection (MOI) of 4 and subsequently treated with six antibiotics at 32× their respective MICs for 4 h. Following treatment, cells were lysed, serially diluted lysates were plated, and intracellular bacterial burden was quantified by CFU enumeration. **(B)** Modulation of the MIC of rifalogue against *S. aureus* LAC in the presence of *t*AIP-III D4A amide at concentrations ranging from 0 to 5 μmol/L.

### 3.2 Design and synthesis of auto-inducing peptide-antibiotic conjugate

We designed and synthesized the QSI-A by covalently linking the AIP-III analog to rifalogue via a cathepsin-cleavable linker (Fig. 2). *t*AIP-III D4A amide was selected as the QS inhibitor based on prior studies demonstrating its potent and broad-spectrum inhibition of all four agr types (I-IV) [18]. The final conjugate, QSI-A, was purified as a hydrochloride salt using reverse-phase high-performance liquid chromatography (RP-HPLC) and structurally characterized by liquid chromatography-mass spectrometry (LC-MS) (Fig. S2).

**Fig. 2:**
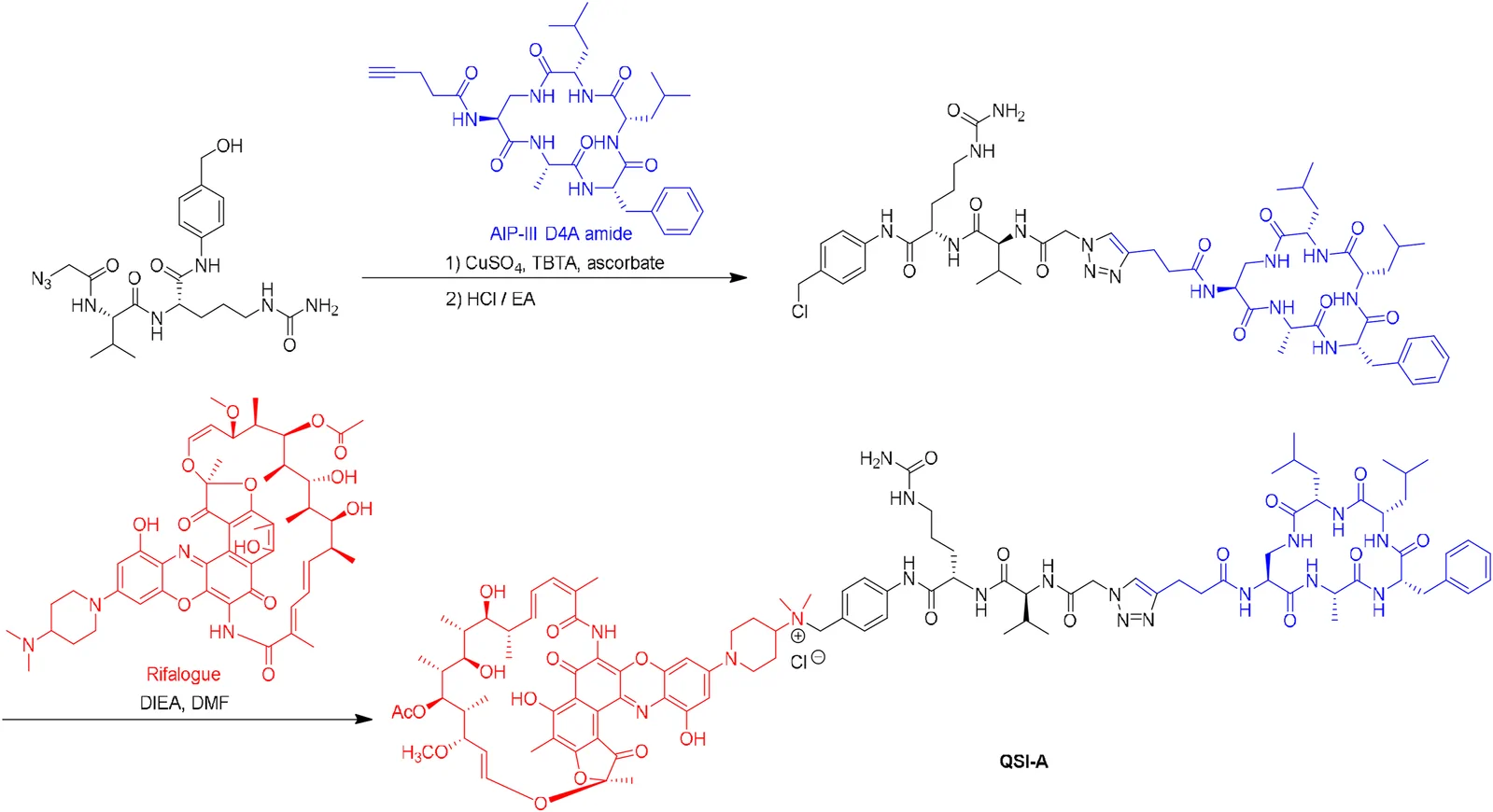
Synthetic scheme showing the conjugation of rifalogue to the autoinducing peptide analog through a cathepsin-cleavable linker.

### 3.3 QSI-A exhibits *in vitro* non-toxicity, controlled antibiotic release, and plasma stability

To evaluate the *in vitro* biocompatibility of QSI-A, we assessed its cytotoxicity against human keratinocyte (HaCaT) cells and its hemolytic activity toward human red blood cells (RBCs). As shown in Fig. 3A, no significant reduction in HaCaT cell viability was observed following treatment with QSI-A at concentrations ranging from 0.5 to 10 μmol/L, relative to the untreated control group; similarly, LDH release remained unchanged under these conditions (Fig. 3B). Moreover, QSI-A displayed no detectable hemolytic activity, even at a concentration of 10 μmol/L, after incubation with 4% human RBCs (Fig. 3C). As anticipated, conjugation of the rifalogue to the *t*AIP-III D4A amide significantly attenuated its extracellular antibacterial activity: the MIC of QSI-A against USA300 LAC was 2.5 μmol/L, compared to 0.03125 μmol/L for the free rifalogue (Table S1). However, upon cathepsin B treatment, the conjugate regained full bactericidal activity, consistent with cleavage-dependent activation (Fig. 3D). Plasma stability represents a critical determinant of therapeutic utility for peptide-antibiotic conjugates, as premature enzymatic degradation or structural modification may impair both their antibacterial efficacy and anti-virulence function. To evaluate the physiological stability of QSI-A, we incubated the conjugate in fresh rat plasma at 37 °C and monitored its antibacterial potency over time using standard MIC assays against USA300 LAC. No statistically significant change in MIC was observed after 3 or 6 h of incubation relative to the baseline (t = 0 h), indicating that QSI-A remains structurally intact and retains its antibacterial activity under physiologically relevant conditions (Fig. 3E).

**Fig. 3:**
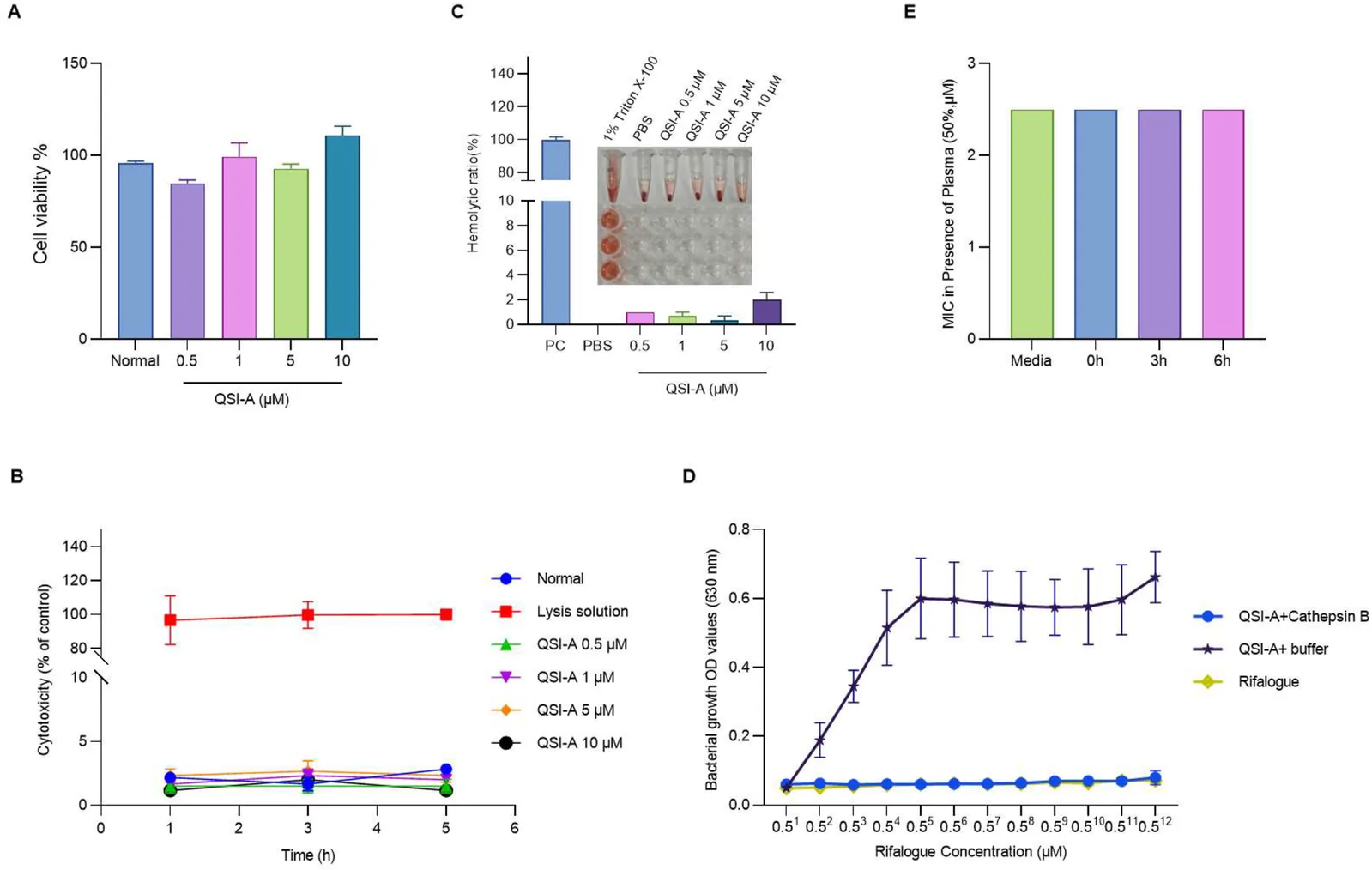
QSI-A exhibits *in vitro* non-toxicity, controlled antibiotic release, and plasma stability. **(A)** Cytotoxicity of QSI-A toward HaCaT cells was assessed using the CCK-8 assay. **(B)** Membrane integrity of HaCaT cells following QSI-A exposure was evaluated via LDH release assay at 1, 3, and 5 h post-treatment. **(C)** Hemolytic activity of QSI-A was determined in a 4% suspension of human red blood cells; 1% Triton X-100 served as the positive control, PC: positive control. **(D)** Cathepsin B-mediated cleavage of QSI-A generates antibacterial activity. **(E)** Plasma stability of QSI-A was evaluated by measuring its residual antimicrobial activity, expressed as MIC, against *S. aureus* LAC in rat blood plasma after incubation for 0, 3, and 6 h. (For interpretation of the references to colour in this figure legend, the reader is referred to the Web version of this article.)

### 3.4 QSI-A inhibits the agr QS system and associated virulence factors in *S. aureus*

To assess the impact of QSI-A on agr QS activity, RT-qPCR was performed to quantify transcript levels of the regulatory gene *agrA*, the effector *RNAIII*, and downstream toxin-encoding genes-*psmα*, *hla*, and *pvl*, that are upregulated by the agr system. In the USA300 LAC strain, treatment with QSI-A at concentrations equal to its MIC markedly suppressed *agrA* and *RNAIII* expression in a concentration-dependent manner relative to the untreated control (Fig. 4A and B). Concurrently, expression of the toxin genes *psmα*, *hla*, and *pvl* were significantly downregulated to varying extents upon QSI-A treatment across tested concentrations (Fig. 4C–E). Furthermore, *δ*-toxin, the protein product translated from *RNAIII*, was quantified in bacterial supernatants using RP-HPLC/MS; results revealed a substantial reduction in *δ*-toxin production in the presence of QSI-A (Fig. 4F). Consistently, bacterial supernatants from QSI-A-treated cultures exhibited markedly diminished hemolytic and cytolytic activities, with complete abrogation observed at higher QSI-A concentrations (Fig. 4G and H). Similar effects were confirmed in the MW2 strain: QSI-A treatment reduced transcription of *agrA*, *RNAIII*, *psmα*, *hla*, and *pvl* (Fig. S4A–E) and attenuated the hemolytic capacity of culture supernatants (Fig. S4F). In addition, we evaluated the effect of QSI-A on biofilm formation. At concentrations up to 1.25 μmol/L, QSI-A did not significantly affect the *in vitro* biofilm-forming ability of *S. aureus* LAC compared to the untreated control (Fig. S5). Collectively, these findings demonstrate that QSI-A retains the agr quorum-quenching activity of *t*AIP-III D4A amide following covalent conjugation to an antibiotic via the linker employed in this study.

**Fig. 4:**
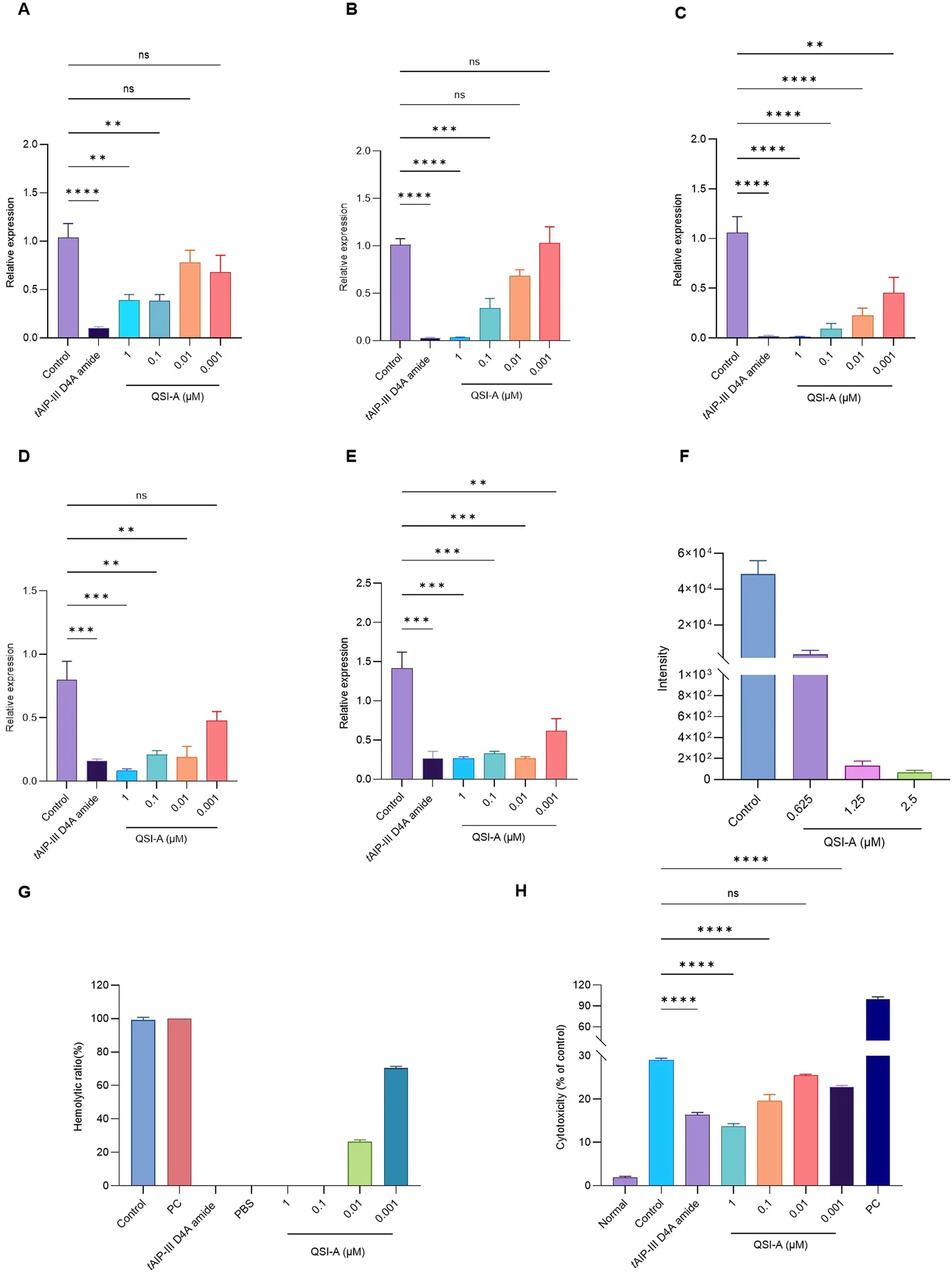
QSI-A inhibits the agr QS system and associated virulence factors in *S. aureus*. **(A-E)** Relative mRNA expression levels of *agrA* (a), *RNAIII* (b), *psmα* (c), *hla* (d), and *pvl* (e) in *S. aureus* LAC following treatment with QSI-A at concentrations of 0.001, 0.01, 0.1, or 1 μmol/L, relative to untreated controls. *t*AIP-III D4A amide (1 μmol/L) was included as a reference agr inhibitor. **(F)** RP-HPLC/MS analysis of *δ*-toxin levels in culture supernatants of *S. aureus* LAC treated with QSI-A at 0.625, 1.25, or 2.5 μmol/L. **(G)** Hemolytic activity of *S. aureus* LAC culture supernatants following QSI-A treatment (0.001, 0.01, 0.1, or 1 μmol/L); PC: positive control. **(H)** Cytolytic activity of *S. aureus* LAC culture supernatants following QSI-A treatment (0.001, 0.01, 0.1, or 1 μmol/L); PC: positive control. Data are presented as mean ± SD of at least three independent experiments. Statistical significance was determined by one-way ANOVA followed by Dunnett's post-hoc test (**, *P* < 0.01; ***, *P* < 0.001; ****, *P* < 0.0001; ns, not significant), comparing all groups to the untreated infected control (or as otherwise indicated).

### 3.5 QSI-A reduces intracellular *S. aureus* burden, limits intercellular dissemination, and suppresses intracellular agr QS

Having confirmed that QSI-A inhibits the agr QS system, we next investigated its intracellular antibacterial activity. QSI-A was applied to a cellular model of intracellular *S. aureus* infection. As shown in Fig. 5A, treatment with 1 μmol/L QSI-A effectively reduced intracellular *S. aureus* within murine macrophage RAW264.7 cells. To determine whether QSI-A also exerts bactericidal activity in non-professional phagocytes, we treated *S. aureus*-infected A549 (lung epithelial), HaCaT (keratinocyte), and MG63 (osteoblast) cell lines. CFU assays revealed that QSI-A significantly reduced intracellular bacterial burdens in all three cell types (Fig. 5B–D). To further assess whether QSI-A inhibits intercellular bacterial dissemination, *S. aureus*-infected peritoneal macrophages were co-cultured with MG63 cells. Addition of QSI-A to the co-culture medium resulted in a significant reduction in viable intracellular bacteria recovered from MG63 cells at 24 h post-infection (Fig. 6). In addition, to assess whether intracellular processing of QSI-A generates antibacterial activity, RAW264.7 macrophages were incubated with QSI-A at 2 μmol/L for 1 h, washed thoroughly to remove extracellular compound, and lysed. As shown in Supplementary Table S3, cell lysates from the untreated control group exhibited no antibacterial activity against *S. aureus* LAC. In contrast, cell lysates derived from QSI-A-treated (2 μmol/L) macrophages displayed clear antibacterial activity, with an MIC value of 25% (i.e., a 1:4 dilution of the lysate). These results provide evidence that QSI-A is internalized by host cells and that intracellular processing of QSI-A generates antibacterial activity, consistent with cathepsin B-dependent activation (Fig. 3D). Consistent with its extracellular agr inhibition, QSI-A also downregulated the expression of *agrA* (Fig. S6A) and *RNAIII* (Fig. S6B) in intracellular *S. aureus*. Moreover, QSI-A effectively eradicated intracellular bacteria in host cells infected with either the community-acquired MRSA strain MW2 (Fig. S7A) or the hospital-acquired MRSA strain N315 (Fig. S7B). Collectively, these findings demonstrate that QSI-A not only efficiently kills intracellular *S. aureus* in both professional and non-professional phagocytes and prevents bacterial spread between host cells *in vitro*, but also suppresses the agr QS system intracellularly.

**Fig. 5:**
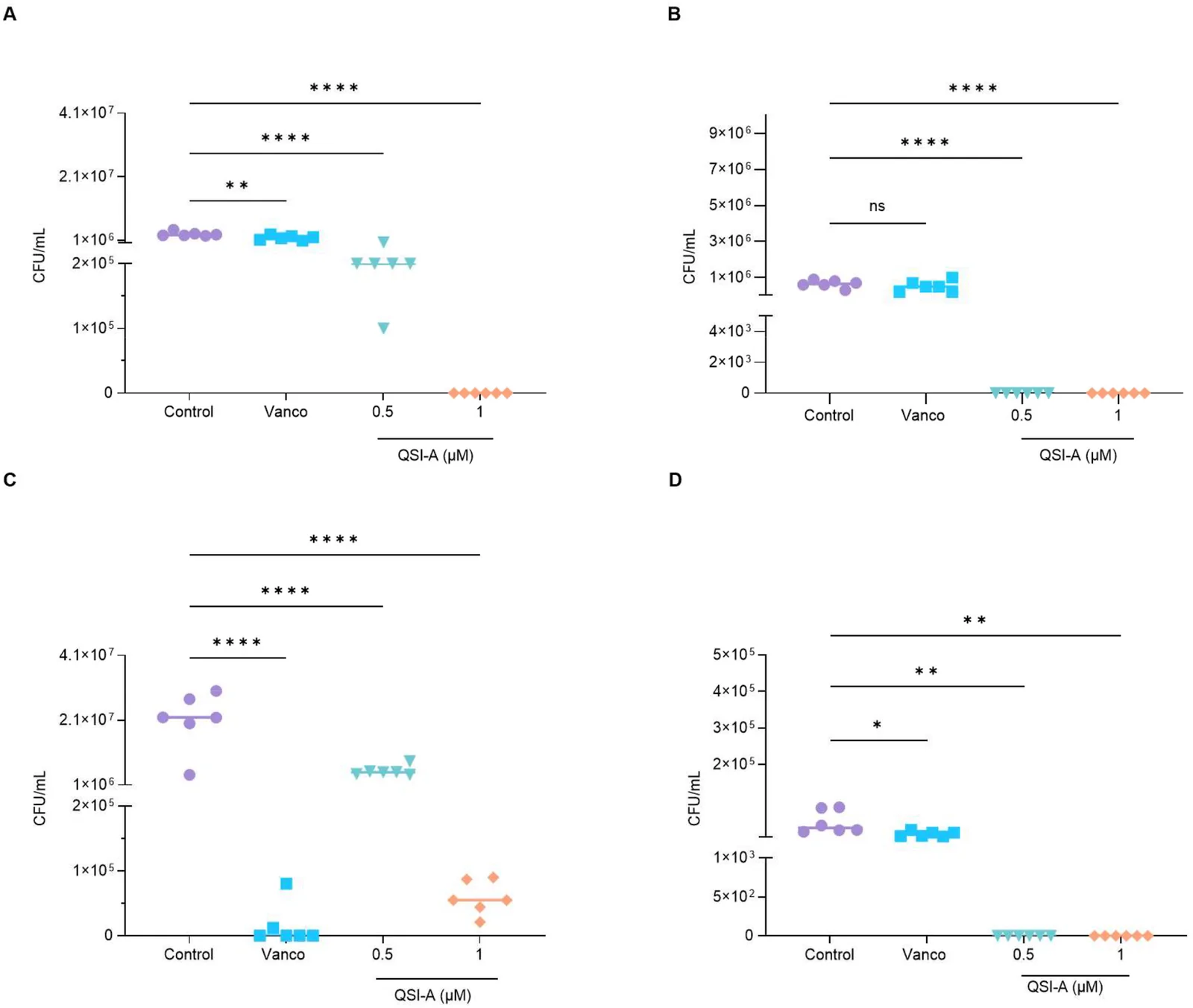
QSI-A reduces intracellular *S. aureus*. RAW 264.7 **(A)**, A549 **(B)**, HaCaT **(C)**, and MG-63 **(D)** cells were infected with *S. aureus* LAC at a multiplicity of infection (MOI) of 4 for RAW 264.7 cells and 10 for A549, HaCaT, and MG-63 cells, and subsequently treated with QSI-A (0.5 or 1 μmol/L) or vancomycin (0.1 mg/mL) for 4 h. Intracellular bacterial burden was quantified by CFU assay. Data are presented as mean ± SD. Statistical significance was determined by one-way ANOVA followed by Dunnett's post-hoc test (n = 6, **, *P* < 0.01; ***, *P* < 0.001; ****,*P* < 0.0001; ns, not significant), comparing all groups to the untreated infected control.

**Fig. 6:**
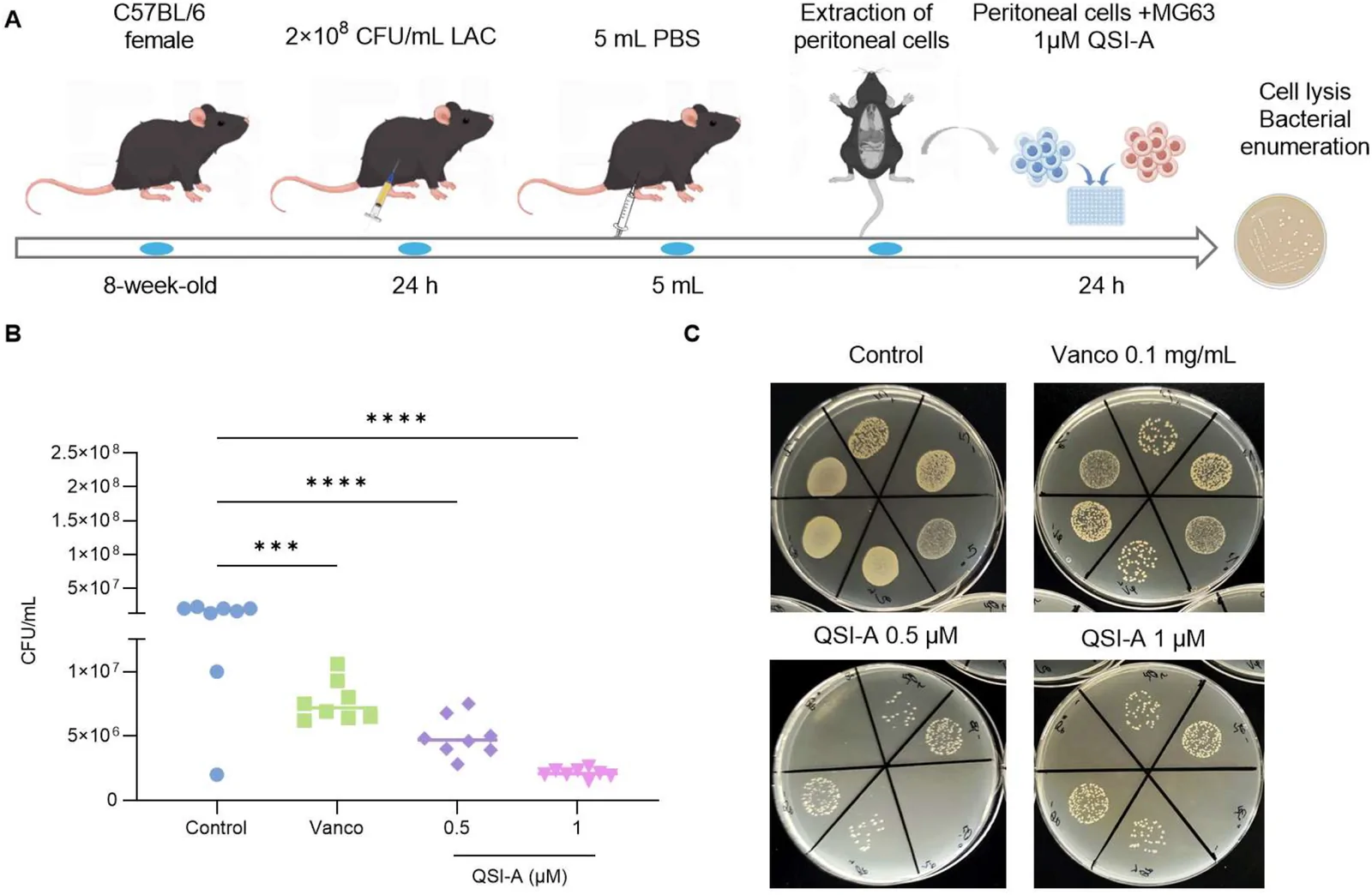
QSI-A inhibits cell-to-cell transmission of *S. aureus*. **(A)** Schematic illustration of the intracellular dissemination assay protocol. **(B)** Quantitative analysis of bacterial counts. **(C)** Representative images of TSA plates used for colony enumeration. Data are presented as mean ± SD. Statistical significance was determined by one-way ANOVA followed by Dunnett's post-hoc test (n = 8, **, *P* < 0.01; ***, *P* < 0.001; ****, *P*< 0.0001), comparing all groups to the untreated infected control.

### 3.6 QSI-A enhances clearance of *S. aureus* in a murine bacteremia infection model

Building upon its promising *in vitro* efficacy, we next evaluated the therapeutic potential of QSI-A *in vivo* using a murine intravenous bacteremia model (Fig. 7). Mice were infected intravenously and subsequently treated 24 h later with a single intraperitoneal dose of QSI-A, vancomycin, rifalogue, or a physical mixture of *t*AIP-III D4A amide and rifalogue. Notably, a single administration of QSI-A led to effective reduction of *S. aureus* from kidney tissue (Fig. 7B&C), accompanied by only mild histopathological changes in renal, lung and liver architecture (Fig. 7D). The physical mixture showed significantly lower *in vivo* antibacterial activity compared to QSI-A, confirming that the covalent conjugation provides a synergistic advantage (Fig. 7B). Countable bacteria were recovered only from kidney homogenates; liver, lung, and blood samples yielded no detectable CFU at the endpoint. QSI-A demonstrated favourable efficacy compared to vancomycin or rifalogue treatment initiated at the same time, although direct PK/PD-equivalent comparisons were not performed.

**Fig. 7:**
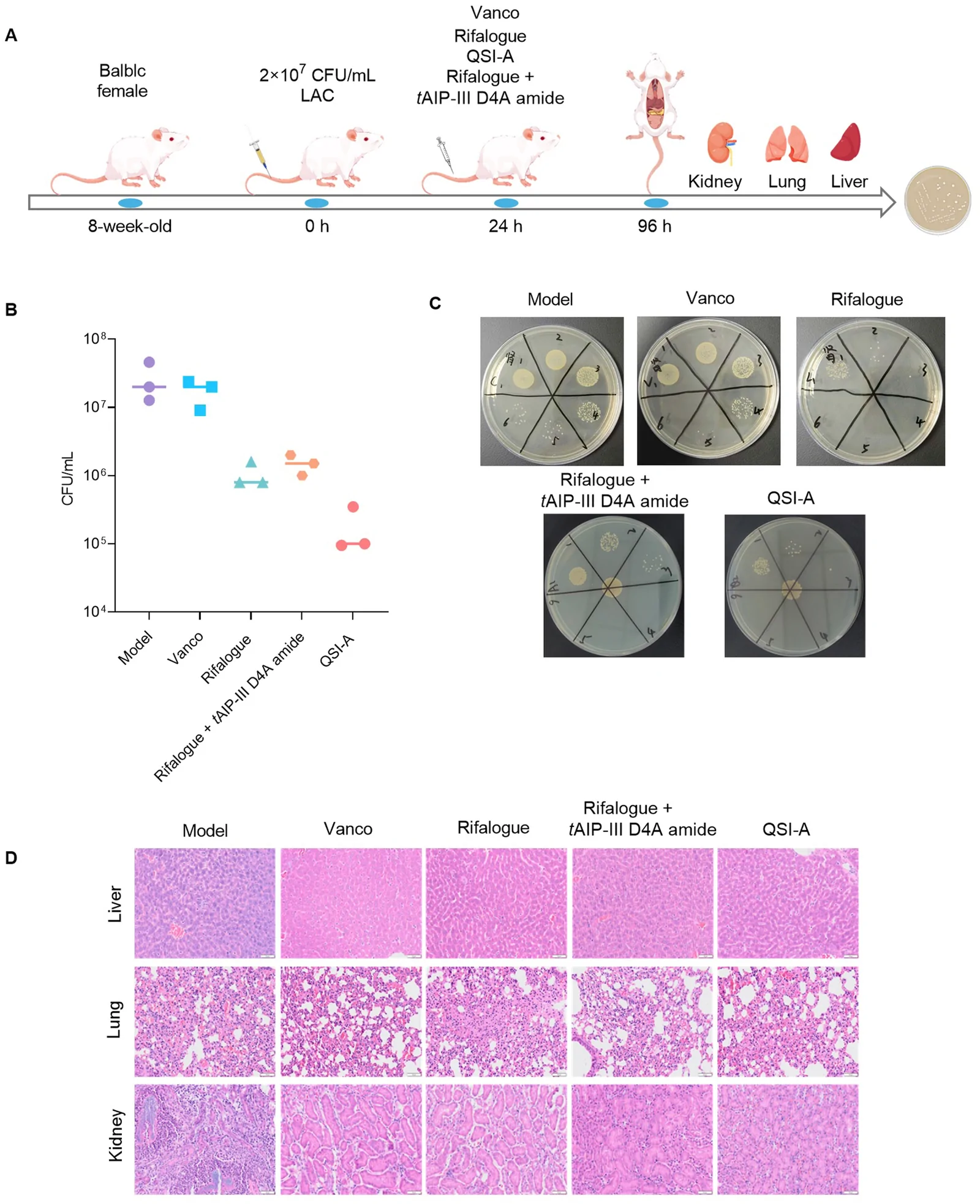
QSI-A promotes *S. aureus* clearance in mouse bacteremia infection model. **(A)** Schematic representation of the experimental workflow in the murine bacteremia model (QSI-A: 1.0 mg/kg; rifalogue: 0.5 mg/kg; vancomycin: 110 mg/kg). **(B)** Bacterial load in murine kidneys. **(C)** Representative TSA plate images for CFU enumeration from kidney homogenates. **(D)** Histopathological analysis of liver, lung, and kidney tissues from infected mice using hematoxylin and eosin (H&E) staining. Scale bar: 50 μm.

### 3.7 QSI-A enters host cells via both direct and indirect mechanisms

To investigate the mechanism by which QSI-A gains entry into host cells, we examined whether it could internalize directly or co-internalize with *S. aureus* by specifically targeting the AgrC receptor on the bacterial surface. Prior to this, we first assessed the cellular uptake efficiency of *t*AIP-III D4A amide by conjugating it with fluorescein isothiocyanate (FITC). Flow cytometry and confocal microscopy analyses revealed no intracellular fluorescence in RAW264.7 cells following incubation with the *t*AIP-III D4A amide-FITC conjugate (Fig. 8A & B), indicating that *t*AIP-III D4A amide alone cannot enter mammalian cells. Next, RAW264.7 cells were pre-incubated with QSI-A for 1 h, after which extracellular QSI-A was thoroughly removed before infection with *S. aureus*. Intracellular survival assays demonstrated that cells pre-treated with QSI-A harbored significantly fewer intracellular *S. aureus* (Fig. 8D). Subsequently, *S. aureus* was pre-incubated with QSI-A for 0.5 h, and these QSI-A-treated bacteria were then used to infect RAW264.7 cells. Consistently, intracellular bacterial loads were markedly reduced in cells infected with QSI-A-pretreated *S. aureus* (Fig. 8E). Moreover, flow cytometry confirmed that the *t*AIP-III D4A amide-FITC conjugate specifically labeled *S. aureus* upon incubation, demonstrating its selective binding to the bacterial surface (Fig. 8C&F). Collectively, these findings indicate that QSI-A exerts intracellular bactericidal activity through dual entry pathways: direct penetration into host cells and co-internalization with *S. aureus* via AgrC receptor targeting.

**Fig. 8:**
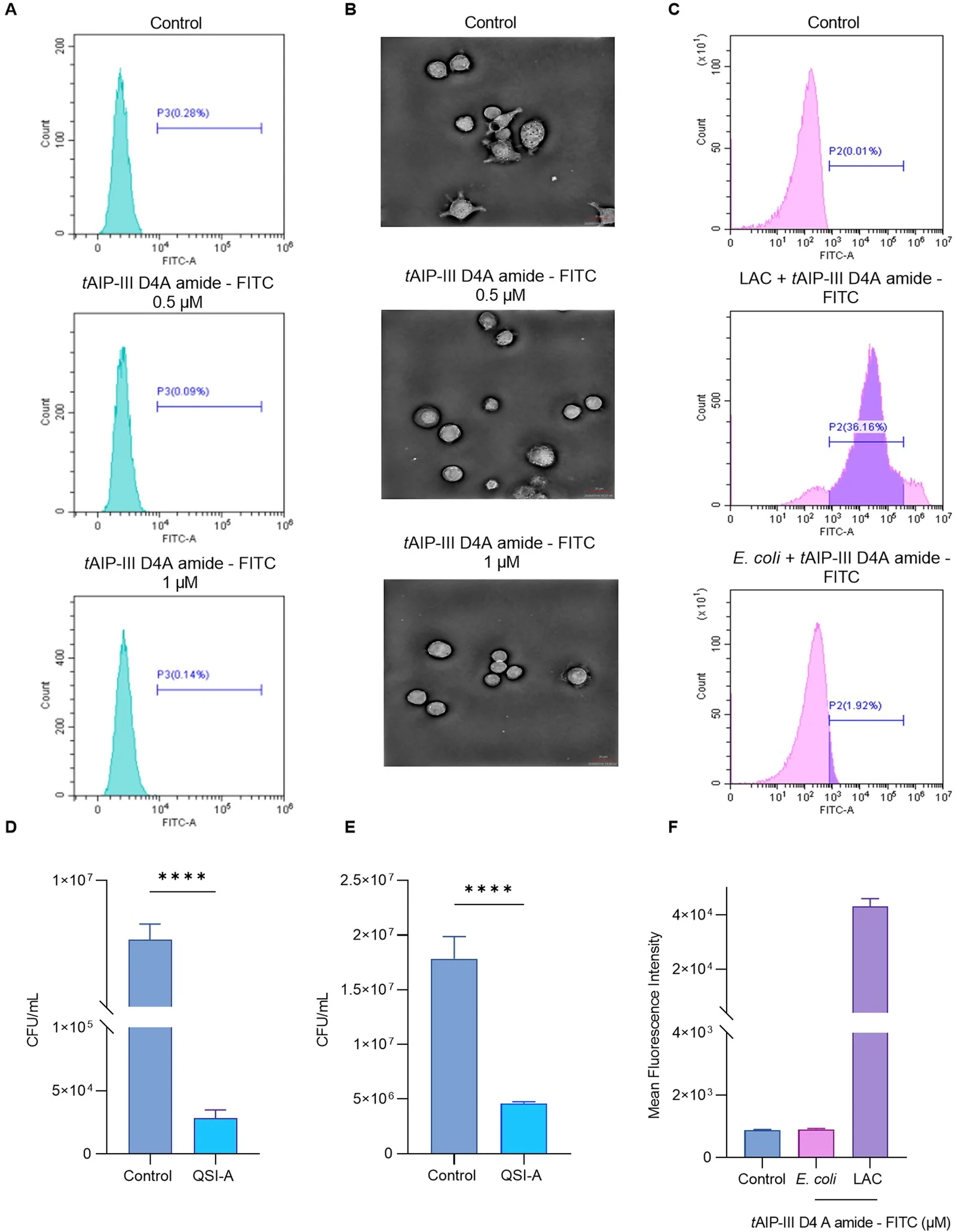
QSI-A enters host cells via both direct and indirect mechanisms. **(A)** Flow cytometry and **(B)** confocal laser scanning microscopy (CLSM) analyses of *t*AIP-III D4A amide-FITC uptake into RAW 264.7 cells at indicated concentrations (0.5 and 1 μmol/L). Scale bar: 20 μm. **(C)** Flow cytometry analysis of FITC-conjugated *t*AIP-III D4A amide (5 μmol/L) binding to wild-type *S. aureus* LAC versus *E. coli* ATCC 25922, and **(F)** the mean fluorescence intensity (MFI) in each group. **(D)** Intracellular bacterial survival in RAW 264.7 cells pre-incubated with QSI-A for 1 h, followed by removal of extracellular QSI-A prior to *S. aureus* LAC infection. **(E)** Intracellular bacterial survival in RAW 264.7 cells infected with *S. aureus* LAC that had been pre-treated with QSI-A for 0.5 h. Data are presented as mean ± SD of at least three independent experiments. Statistical significance was determined by Student's *t*-test (**, *P* < 0.01; ***, *P*< 0.001; ****, *P* < 0.0001; ns, not significant), comparing between the two indicated groups.

## 4 Discussion

Virulence factors and intracellular survival represent two key determinants underlying the pathogenesis of *S. aureus*. It is well established that the expression of numerous virulence factors in *S. aureus* is tightly regulated by the agr QS system [19]. Consistent with this, agr mutant or knockout strains induce significantly milder infections compared to the wild-type strain across multiple animal infection models [20]. Emerging evidence increasingly demonstrates that *S. aureus* can persist and replicate within a broad range of host cells, including both professional phagocytes and non-phagocytic cells [[21], [22], [23], [24], [25]], thereby exploiting these cells as “Trojan horses” to evade antimicrobial agents and host immune surveillance, while facilitating cell-to-cell bacterial dissemination [26,27]. Intracellular persistence of *S. aureus* is now widely recognized as a major contributor to chronic, relapsing, and treatment-refractory infections [28,29]. To simultaneously attenuate virulence and impair intracellular survival, we herein developed a novel therapeutic strategy: a QS inhibitor-antibiotic conjugate (QSI-A), constructed from an AIP derivative. This peptide-antibiotic conjugate is designed to (i) disarm bacterial virulence by specifically inhibiting the agr QS system, and (ii) undergo intracellular processing to exert potent intracellular bactericidal activity. Our results demonstrate that QSI-A effectively suppresses agr-mediated QS signaling and concomitantly attenuates virulence factor expression. Moreover, QSI-A significantly reduces intracellular survival of *S. aureus* across diverse host cell types. Most notably, a single dose of QSI-A achieves superior therapeutic efficacy in a murine model of bacteremia, showing favourable activity compared to vancomycin and free rifalogue in this preliminary study.

To select an appropriate antibiotic, we first evaluated the intracellular antibacterial activity of several antibiotics commonly used in clinical practice for *S. aureus* infection, including vancomycin, tetracycline, imipenem, cefoperazone, and rifampicin, as well as rifalogue, a novel rifamycin derivative. Our results demonstrated that most currently available antibiotics fail to effectively eradicate intracellular *S. aureus*, corroborating prior reports of the intrinsic resistance of intracellular *S. aureus* to conventional antibiotic therapy [30,31]. Although rifalogue has not yet entered clinical use, previous studies have established its potent antibacterial activity against both planktonic and intracellular *S. aureus*, and it has been employed as the cytotoxic payload in antibody-antibiotic conjugates (AACs) designed specifically to eliminate intracellular *S. aureus* [27,32]. Consistent with these earlier findings [27], our data confirm that rifalogue exhibits superior intracellular antibacterial activity against *S. aureus* across multiple host cell types. Rifamycins are distinguished by their stability under acidic conditions-enabling them to withstand the harsh intracellular environment, particularly within phagolysosomes [31]. Furthermore, rifalogue effectively eliminates non-replicating bacteria and antibiotic-tolerant persister cells, and readily accumulates within the intracellular compartment [27]. Notably, we observed that rifalogue's antibacterial efficacy is significantly enhanced in the presence of the agr QS inhibitor *t*AIP-III D4A amide, a phenomenon attributable to the recently identified agr-regulated rifampicin efflux system [33]. Collectively, these properties, along with our experimental findings, strongly support rifalogue as a highly promising candidate for the treatment of intracellular *S. aureus* infections, with its therapeutic potential further augmented upon pharmacological inhibition of the agr QS system.

*S. aureus* employs AIPs to activate and amplify its agr QS system, primarily through binding to the AgrC receptor on the bacterial surface. Capitalizing on this AIP-AgrC interaction, various synthetic AIP analogues have been developed to inhibit the agr system by competitively antagonizing native AIP binding [34]. Among these, the analog *t*AIP-III D4A amide has shown particularly broad and potent inhibition across all four agr types [15]. Recently, Kyoji et al. demonstrated that truncated AIP scaffolds can serve as targeting vehicles: when conjugated to cytotoxic photosensitizers, they enhance antibacterial efficacy by delivering more photosensitizer molecules to the bacterial surface via AgrC binding [17]. Beyond functioning as delivery carriers for antibacterial agents, AIP analogues have also been fused to antimicrobial peptides, enabling dual functionality-simultaneously inhibiting the agr QS system and exerting direct antibacterial activity [35]. In our study, we conjugated *t*AIP-III D4A amide to a rifalogue using a protease-sensitive linker, generating a hybrid molecule termed QSI-A. Compared with *t*AIP-III D4A amide alone, QSI-A effectively suppressed agr-mediated QS and reduced *S. aureus* virulence, confirming that AIP analogues can be functionally fused to other molecular entities without losing their agr-inhibitory activity, consistent with earlier reports [17].

Biofilm formation is another phenotype that can be influenced by the agr QS system, though the relationship is complex and context-dependent. In our study, QSI-A showed only a modest reduction in biofilm formation at the highest sub-MIC concentration tested (1/2 MIC), with no significant effect at lower concentrations. Notably, since agr activation is generally associated with biofilm dispersal, inhibition of agr would not be expected to reduce biofilm formation. Therefore, the observed minor effect at 1/2 MIC is unlikely to be mediated through agr inhibition and may instead reflect non-specific physicochemical effects of the conjugate at higher concentrations, such as altered bacterial surface properties or initial adhesion. Importantly, this effect was weak, concentration-limited, and did not manifest at lower concentrations, indicating that biofilm modulation is not a primary mechanism of QSI-A. The principal anti-infective actions of QSI-A remain the suppression of agr-mediated virulence factors and the targeted elimination of intracellular bacteria.

Except for its poor antibacterial activity under intracellular conditions, the limited cellular penetration of conventional antibiotics, particularly into host cells, also contributes significantly to their inefficacy against intracellular bacteria. To address the clinical threat posed by intracellular *S. aureus* infection, two primary delivery-strategy paradigms have been developed, both centered on engineered systems designed to target intracellular bacteria: one strategy focuses on targeting host cells, while the other targets the bacteria themselves. In the host-cell-targeting approach, antibiotics are either encapsulated within micro- or nanocarriers or covalently conjugated to membrane-permeable active molecules, thereby enhancing cellular membrane penetration and subsequent intracellular uptake [[36], [37], [38]]. In contrast, the bacteria-targeting strategy employs antibody-antibiotic conjugates (AACs), which bind specifically to bacterial surface antigens and, upon Fcγ receptor-mediated endocytosis into host cells, release the active antibiotic within the intracellular proteolytic environment [27,39]. Based on the robust intracellular antibacterial activity of QSI-A against *S. aureus* observed in our study, we further investigated its underlying mechanistic basis. As anticipated, conjugation of the rifalogue to *t*AIP-III D4A amide markedly attenuated its antibacterial potency: the MIC against the USA300 LAC strain increased from 0.03125 μmol/L to 2.5 μmol/L. Notably, all experiments were conducted using QSI-A concentrations ≤1 μmol/L-a range confirmed to exert no direct bactericidal effect on *S. aureus*. First, to assess whether *t*AIP-III D4A amide itself could directly penetrate host cells, we labeled it with FITC and evaluated its cellular uptake. Confocal imaging and flow cytometry analyses confirmed that *t*AIP-III D4A amide-FITC failed to enter host cells, indicating that AIP analogues are unlikely to serve as intrinsic carriers for co-delivering other molecules across the plasma membrane, a finding consistent with a previous study [17]. Subsequently, we pre-incubated host cells with QSI-A, thoroughly washed and replaced the culture medium, and then infected the cells with *S. aureus*. Strikingly, we observed a significant reduction in intracellular bacterial burden, despite the absence of direct antibacterial activity at the applied QSI-A concentration, suggesting that QSI-A efficiently enters host cells and releases its active antibiotic payload intracellularly. Furthermore, we employed *S. aureus* pre-incubated with QSI-A prior to host cell infection and observed a significant reduction in the intracellular survival of *S. aureus*. This finding indicates that QSI-A can specifically target *S. aureus* and co-internalize with the bacteria into host cells, followed by localized generation of antibacterial activity, mirroring the mechanism of action of antibody-antibiotic conjugates (AACs). Collectively, these results demonstrate that QSI-A exerts its antibacterial effect through intracellular processing that generates an antibacterial species, either after direct cellular penetration or via co-internalization with bacteria, mediated by specific targeting of the AgrC receptor.

Regarding the distinct vancomycin effect observed in HaCaT cells (Fig. 5C), we interpret this cell-type-dependent phenomenon as a result of differential intracellular accumulation of vancomycin across host cell lines. As non-professional phagocytes, HaCaT cells internalize bacteria and antibiotics primarily via endocytosis, and the efficiency of this process, along with intracellular drug efflux pump activity, may vary considerably among cell types. In HaCaT cells, vancomycin may accumulate to a threshold sufficient for intracellular bacterial killing, whereas in macrophages or other epithelial cells, lower endocytic uptake or higher efflux could result in sub-therapeutic concentrations. Importantly, the primary purpose of including multiple cell lines was to validate the broad-spectrum intracellular activity of QSI-A, rather than to compare absolute efficacy of vancomycin. QSI-A consistently reduced intracellular bacterial burdens in all tested cell lines.

## 5 Limitations

Several limitations of this study warrant consideration. First, the molecular mechanism underlying QSI-A's autonomous cellular uptake remains incompletely understood. Although our experimental data show that the AIP analogues alone cannot enter host cells, the QSI-A conjugate, comprising AIP linked to rifalogue, readily gains intracellular access. This suggests that rifalogue plays a critical role in facilitating QSI-A's autonomous cellular entry, potentially attributable to the inherent lipophilicity of rifamycin-class antibiotics [40]. In addition, the present study does not directly quantify intracellular QSI-A or free rifalogue concentrations. Although these data support the intracellular generation of antibacterial activity, the chemical identity of the active species was not directly determined. Therefore, the intracellular exposure, stability, and processing profile of QSI-A remain to be fully defined. Second, from a translational perspective, QSI-A may face practical constraints in clinical application. Since certain clinically relevant *S. aureus* infections are caused by agr-negative strains [41,42], which lack functional AgrC expression; consequently, QSI-A cannot effectively target such strains, thereby limiting its therapeutic breadth. Third, the *in vivo* efficacy data should be interpreted with caution. The doses of QSI-A, rifalogue, and vancomycin used in the murine bacteremia model were selected based on previously reported effective doses rather than on strict pharmacokinetic/pharmacodynamic (PK/PD) equivalence, which limits direct comparisons of their relative potency. In addition, the sample size in each treatment group was small (n = 3), and although the results are encouraging, they should be considered preliminary and warrant further validation in larger cohorts with optimized dosing regimens.

## 6 Conclusion

In conclusion, QSI-A represents a promising dual-functional strategy for combating *S. aureus* infection: it attenuates bacterial virulence through potent inhibition of the agr QS system while simultaneously enabling targeted reduction of intracellular bacterial reservoirs via partly receptor-directed intracellular activation. As such, the QS inhibitor-antibiotic conjugate platform holds considerable potential not only for *S. aureus* but also as a promising approach for treating other highly virulent, intracellular pathogens.

## CRediT authorship contribution statement

**Meiling Ma:** Writing – original draft, Validation, Formal analysis. **Shihao Luo:** Writing – original draft, Data curation. **Junshu Wang:** Methodology, Formal analysis. **Zhuang Nie:** Validation. **Shaonan An:** Validation. **Jianyu Wang:** Methodology. **Xiangxiang He:** Data curation. **Fei Da:** Writing – review & editing, Resources. **Lin Yao:** Writing – review & editing, Project administration, Funding acquisition.

## Ethics approval

All the animal studies were performed in accordance with the national regulations on animal studies. The animal experimental protocols were approved by the medical ethics committee of the Fourth Military Medical University (No. KY20212479-C-1).

## Data availability

All data supporting the findings of this study are included in this published article and its supplementary information files.

## Declaration of generative AI in scientific writing

Not applicable.

## Funding information

The funding support from the 10.13039/501100001809National Natural Science Foundation of China (82102445, 82202571), State Key Laboratory of New Targets Discovery and Drug Development for Major Diseases (SKLD2025Q01) , Key Project of Shaanxi Provincial Natural Science Basic Research Program (2026SYS-SYSZD-48) and the 10.13039/501100007547Fourth Military Medical University (2024JC051, 2023KXKT031) are gratefully acknowledged.

## Declaration of competing interest

The authors declare that they have no known competing financial interests or personal relationships that could have appeared to influence the work reported in this paper.
