# Supplementary material for: Quorum sensing inhibitor-antibiotic conjugate suppresses virulence and eradicates intracellular Staphylococcus aureus
Source: Pharm Sci Adv. 2026 Sep 28;4:100155. doi: 10.1016/j.pscia.2026.100155 (PMC13694733; doi:10.1016/j.pscia.2026.100155)
Supplement: Supplementary file 1 [file mmc1.docx]

Supporting Information

Quorum Sensing Inhibitor-Antibiotic Conjugate Suppresses Virulence and Eradicates Intracellular *Staphylococcus aureus*

Meiling Ma^1,2#^, Shihao Luo^1,2#^, Junshu Wang^3#^, Zhuang Nie^1^, Shaonan An^1^, Jianyu Wang^4^, Xiangxiang He^1^, Fei Da^3*^, Lin Yao^1*^

^1^Department of Medicinal chemistry and Pharmaceutical Analysis, School of Pharmacy, Fourth Military Medical University, Xi’an, P. R. China

^2^Department of Pharmacy, Shaanxi University of Chinese Medicine, Xianyang 712046, P. R. China

^3^Military Medical Innovation Center, Fourth Military Medical University, Xi’an, 710032, P. R. China

^4^Department of Radiation Medical Protection, School of Military Preventive Medicine, Fourth Military Medical University, Xi'an, P. R. China

^#^These authors contribute equally to this work.

*Corresponding authors: Fei Da, email address: [dayangfei1988@163.com](mailto:dayangfei1988@163.com); Lin Yao, email address: yaolinlin@fmmu.edu.cn

**CONTENTS**

**Experimental Methods**

1. Chemicals and reagents……………………………………………………………….S3
2. Synthesis of the QSI-A……………………………………………………………......S3
3. Bacterial strains…………………………………………………………………........S5
4. Cell culture…………………………………………………………………...............S5
5. Determination of Minimum Inhibitory Concentration (MIC)…………………................S5
6. Cell Counting Kit-8 (CCK-8) assay…………………...............................................S5
7. Hemolysis assay………………….............................................................................S6
8. Cytotoxicity assay (LDH release)…………………..................................................S6
9. Cathepsin release assay for QSI-A………………….................................................S7
10. Plasma stability assay…………………...................................................................S7
11. RT-qPCR…………………......................................................................................S8
12. Detection of *δ*-toxin in bacterial culture supernatants…………………..................S8
13. Biofilm formation assay….......................................................................................S9
14. Intracellular *S. aureus* killing assay…………………..............................................S9
15. Intracellular antibiotic release assay……………….................................................S9
16. Intercellular dissemination assay…………………..................................................S10
17. Mouse bacteraemia infection model………………….............................................S10
18. Flow cytometry study…………………...................................................................S10
19. Confocal live-cell Imaging…………………...........................................................S11
20. Statistical analysis…………………........................................................................S11

**Supporting Figures**

Figure S1: Analytical LC-MS chromatogram of the chlorobenzyl intermediate 4……………S12

Figure S2: Analytical LC-MS chromatogram of the target product QSI-A…………………..S13

Figure S3: Analytical HPLC chromatogram of the target product QSI-A…………………....S14

Figure S4. QSI-A inhibits the agr QS system and associated virulence factors in MW2……...S15

Figure S5. The influence of QSI-A on LAC biofilm production.............................................S16

Figure S6. Suppression of agrA and RNAIII in intracellular *S. aureus* LAC by QSI-A………S16

Figure S7. QSI-A eliminates intracellular *S. aureus* MW2 and N315 in RAW264.7 cells……S17

Table S1. Minimum Inhibitory Concentration (MIC) of antibiotics in *S. aureus* LAC……….S17

Table S2. The sequences of primers used in this study…………………..............................S18

Table S3. MIC of cell lysates from QSI-A treated macrophages against *S. aureus* LAC……..S18

References…………………...............................................................................................S19

**Experimental Methods.**

1. **Chemicals and reagents.**

All chemicals were used as received without further purification. The peptide and the linker were purchased from the Hangzhou Specialized Peptide Biotechnology Co., Ltd in China. Sodium ascorbate, copper(II) sulfate, sodium sulfate, hydrochloric acid (HCl), *N*,*N*-Diisopropylethylamine (DIPEA) were obtained from TCI and Energy Chemical. Reagent-grade solvents-dichloromethane (DCM), N,N-dimethylformamide (DMF), methanol, and were pursed from Sigma-Aldrich. Cell culture reagents included High-glucose Dulbecco's Modified Eagle Medium (DMEM) from Gibco, fetal bovine serum (FBS) from Biological Industries, and Phosphate-buffered saline (PBS) from SRL. Tryptic Soy Broth (TSB) and Tryptic Soy Agar (TSA) were purchased from OXOID. The 1% Triton X-100 solution was obtained from Beyotime. Molecular biology reagents comprised the Cell Total RNA Isolation Kit (FOREGENE, China), 5×All-In-One RT MasterMix (abm, Canada), and BlasTaq™ 2× qPCR MasterMix (abm, Canada). The FITC-conjugated targeting peptide was custom-synthesized. Analytical and preparative reverse-phase HPLC was performed using a Kinetex 2.6 µm C18 100Å (100 × 2.1 mm) column (Phenomenex) with water and acetonitrile (containing 0.1% TFA) as the mobile phase.

2. **Synthesis of the QSI-A**

**Scheme S1**. Synthetic scheme of the QSI-A.

***Step I: Preparation of the linker-peptide conjugate***

The linker **1** (68 mg, 0.147 mmol) and the peptide **2** (90 mg, 0.147 mmol) were added to a reaction vessel, followed by methanol (6 mL), water (0.5 mL), sodium ascorbate (VcNa, 58 mg, 0.295 mmol), and copper(II) sulfate (CuSO₄, 11.7 mg, 0.073 mmol). The reaction was allowed to proceed at room temperature for 1h. LC-MS analysis indicated completion of the reaction. Water (10 mL) was added to the reaction mixture, which was then extracted with ethyl acetate:isopropyl alcohol (4:1, 20 mL × 3). The combined organic layers were washed with water (10 mL × 2), dried over anhydrous sodium sulfate, and concentrated under reduced pressure to afford 150 mg of an off-white solid **3**. The yield was not calculated due to excess mass.

***Step II: Conversion of the benzyl alcohol to benzyl chloride***

Add HCl/EA (5 mL) to **3** (130 mg, 0.12 mmol) at room temperature. The reaction mixture was stirred at room temperature for 3h. After concentration under reduced pressure, the residue was repeatedly co-evaporated with dichloromethane (5 times) to remove residual hydrogen chloride, affording the target product **4** (140 mg, pale yellow solid, crude) without yield calculation. LC-MS (ESI) m/z: 1091.6 [M+H]⁺.

***Step III: Congugate of the linker-peptide with the rifalogue***

To a solution of **4** (105 mg, 0.0972 mmol) in DMF (3 mL) cooled in an ice bath was added a solution of the rifalogue **5** (30 mg, 0.0324 mmol) in DMF (1 mL). The reaction mixture was stirred at room temperature for 30min. Then, a solution of DIPEA (21.3 mg, 0.162 mmol) in DMF (1 mL) was added, and the mixture was stirred at 60 ^o^C for 16h. The crude mixture was purified directly by reverse-phase preparative HPLC (MeCN/H₂O with 0.1% formic acid) and lyophilized to afford the target product QSI-A (10 mg, 15.4% yield) as a dark blue solid. LC-MS (ESI) m/z: 1982.8 [M]^+^.

**3. Bacterial strains**

*Staphylococcus aureus* strains were cultured in Tryptic Soy Broth (TSB) or on Tryptic Soy Agar (TSA) (Oxoid, UK). Strain USA300 LAC was kindly provided by Dr. Michael Otto (Laboratory of Human Bacterial Pathogenesis, NIAID, NIH, USA). Strains N315 and MW2 were gifts from Dr. Yujie Li (Department of Life Science and Medicine, University of Science and Technology of China, Hefei, China). Strains Mu50, Escherichia coli ATCC 25922, and *S. aureus* ATCC 29213 were obtained from our laboratory collection.

**4. Cell culture**

Murine monocyte-macrophage leukemia cells (RAW264.7), human non-small cell lung carcinoma cells (A549), human immortalized keratinocytes (HaCaT), and human osteosarcoma cells (MG63) were all obtained from the Cell Engineering Research Center (Fourth Military Medical University, Xi'an, China). Unless otherwise stated, cells were maintained in high-glucose Dulbecco’s Modified Eagle Medium supplemented with 10% fetal bovine serum, and incubated at 37 °C in a humidified atmosphere containing 5% CO₂.

**5. Determination of Minimum Inhibitory Concentration (MIC)**

The minimum inhibitory concentration (MIC) against extracellular *S. aureus* was determined using the broth microdilution method. Briefly, serial two-fold dilutions of QSI A were prepared in tryptic soy broth (TSB) in 96 well plates, with each concentration tested in quadruplicate. An exponential phase culture of the methicillin resistant *S. aureus* strain USA300 LAC was adjusted to approximately 1 × 10^6^ CFU/mL in TSB. A fixed volume of this bacterial suspension was added to each well containing the compound dilutions. After incubation for 18-24 h at 37 °C with shaking, bacterial growth was assessed by measuring the absorbance at 600 nm. The MIC was defined as the lowest concentration of QSI A that inhibited visible bacterial growth by ≥90 % compared to the untreated control.

**6. Cell Counting Kit-8 (CCK-8) assay**

Cell viability was evaluated using a Cell Counting Kit 8 (CCK 8) assay. Cells in the exponential growth phase were harvested and seeded into 96 well plates at a density of 2 × 10⁴ cells per well in 100 μL of complete medium. After 24 h of incubation (37 °C, 5% CO_2_) to allow cell adhesion, the medium was replaced with 100 μL of serum free medium containing QSI A at final concentrations of 0.5, 1, 5, and 10 μmol/L. A vehicle control (0.1% DMSO) was included in each experiment. Following 24 h of exposure under standard culture conditions, 10 μL of CCK 8 reagent was added to each well, and the plates were further incubated for 1-4 h at 37 °C in the dark. Absorbance was measured at 450 nm using a microplate reader. All treatments were performed in triplicate, and cell viability was expressed as a percentage relative to the vehicle control.

**7. Hemolysis assay**

**7.1 Direct hemolytic toxicity of QSI-A**

Human erythrocytes were isolated from heparinized whole blood (10 mL) collected from a healthy volunteer. The blood was centrifuged at 500 × g for 5 min at room temperature, and the supernatant was discarded. The erythrocyte pellet was resuspended with an equal volume of phosphate‑buffered saline (PBS, pH 7.4) and washed by repeating the centrifugation step (500 × g, 5 min) at least three times, until the supernatant appeared clear. The washed erythrocytes were finally resuspended in PBS to obtain an 8% (v/v) suspension.

For hemolysis testing, 200 μL of the erythrocyte suspension was mixed with 200 μL of serially diluted QSI‑A solutions (prepared in PBS) and incubated at 37 °C for 1 h. Controls included: a negative control (200 μL erythrocyte suspension + 200 μL PBS) and a positive control (200 μL erythrocyte suspension + 200 μL of 1% (v/v) Triton X‑100). After incubation, the mixtures were centrifuged at 300 × g for 5 min at 4 °C. A 100 μL aliquot of each supernatant was transferred to a 96‑well plate, and absorbance was measured at 405 nm using a microplate reader. The hemolysis percentage was calculated according to the following formula:

Hemolysis (%) = [(OD sample –OD negative control)/( OD positive control−OD negative control)]×100

**7.2 Hemolytic activity of supernatant from QSI-A-treated bacteria**

*S. aureus* LAC (1 × 10⁶ CFU/mL) was incubated with or without QSI‑A for 8 h at 37 °C with shaking at 220 rpm. The culture supernatant was collected by centrifugation at 12,000 rpm for 10 min at 4 °C. A 200 μL aliquot of the supernatant was mixed with 200 μL of the 8% erythrocyte suspension and incubated at 37 °C for 1 h. Subsequent centrifugation, absorbance measurement, and calculation of hemolysis percentage were performed as described in Section 7.1.

**8.** **Cytotoxicity assay (LDH release)**

**8.1 Direct cytotoxic effect of QSI-A**

The direct cytotoxic effect of QSI A was evaluated in HaCaT cells by measuring lactate dehydrogenase (LDH) release. Cells were seeded in a 96 well plate at a density of 5 × 10⁴ cells per well and allowed to adhere overnight. Subsequently, cells were treated with QSI A at final concentrations of 0.5, 1, 5, and 10 μmol/L, prepared by serial dilution of a 2.4 mM stock solution in culture medium. Controls included a vehicle control (0.4% DMSO), a positive control (cells lysed with 10 μL of lysis solution), and blank wells containing medium only. After incubation for 1, 3, or 5 h, 50 μL of supernatant from each well was transferred to a new 96 well plate, mixed with an equal volume of LDH assay working solution, and incubated in the dark for 30 min. The reaction was stopped by adding 50 μL of stop solution, and absorbance was measured at 490 nm. Cytotoxicity was calculated as follows:

Cytotoxicity (%) = [(OD sample – OD blank) / (OD positive control – OD blank)] × 100

**8.2 Cytotoxicity of supernatant from QSI‑A‑treated bacteria**
To assess the cytotoxicity of bacterial metabolites modulated by QSI‑A, culture supernatant from treated *S. aureus* LAC was collected and applied to HaCaT cells. Briefly, LAC (1 × 10⁶ CFU/mL) was incubated with various concentrations of QSI‑A for 8 h at 37 °C with shaking at 220 rpm. The bacterial culture was centrifuged at 12,000 rpm for 10 min, and the supernatant was collected. HaCaT cells, prepared and seeded as described in Section 8.1, were then incubated with this bacterial supernatant for 1 h. After incubation, LDH activity in the cell culture supernatant was measured, and cytotoxicity was calculated using the same procedure outlined in Section 8.1.

**9. Cathepsin release assay for QSI-A**

To assess the enzymatic release of active antibiotic from QSI‑A, the compound was diluted to 200 μg/mL in cathepsin buffer (20 mM sodium acetate, 1 mM EDTA, 5 mM L‑cysteine, pH 5.0). Cathepsin B (from bovine spleen, Sigma‑Aldrich, C7800) was added to the solution at a final concentration of 10 μg/mL, followed by incubation at 37 °C for 1 h. A control sample was prepared under identical conditions but without the addition of enzyme (QSI‑A in buffer only).

The reaction was terminated by adding nine volumes of tryptic soy broth (TSB, pH 7.4). To quantify the amount of released active antibiotic, serial dilutions of the reaction mixture were prepared in TSB in quadruplicate in a 96‑well plate. Methicillin‑resistant Staphylococcus aureus USA300 (MRSA) was added to each well at a final density of 2 × 10³ CFU/mL. After overnight incubation at 37 °C with shaking, bacterial growth was determined by measuring the absorbance at 630 nm using a microplate reader.

10. **Plasma stability assay^[1]^**

Rat plasma was obtained from Sprague-Dawley rats (male, 8-10 weeks old, 200-250 g) housed at the Laboratory Animal Center of the Fourth Military Medical University. Blood was collected via cardiac puncture under anesthesia, and plasma was separated by centrifugation (3,000 rpm, 5 min). All procedures were approved by the ethics committee of the Fourth Military Medical University (Approval no. KY20212479-C-1).

Rat plasma was prepared by centrifuging freshly collected blood samples at 3,000 rpm for 5 min. To evaluate the stability of QSI A in plasma, the compound was incubated at 37 °C in a mixture containing 50% (v/v) rat plasma and 50% (v/v) bacterial culture medium (tryptic soy broth, TSB). Aliquots were collected at predetermined time points (0, 3, and 6 h). After each incubation interval, the remaining antibacterial activity of QSI A was determined by measuring its minimum inhibitory concentration (MIC) against *S. aureus* LAC using the broth microdilution method described in Section 5. The change in MIC over time served as an indicator of the compound’s stability in plasma.

**11. RNA extraction and quantitative Real‑Time PCR (RT‑qPCR)**

**11.1 Bacterial culture and drug treatment**
Bacterial cultures were grown to mid log phase (OD600 = 0.5–0.6), adjusted to 0.5 McFarland units, and then 75 μL of the suspension was mixed with an equal volume of QSI A solution in a 96 well plate to achieve final concentrations of 1, 0.1, 0.01, and 0.001 μmol/L. The plate was incubated at 37 °C in a humidified chamber for 5 h. Phosphate buffered saline (PBS) was used as a vehicle control.

**11.2 RNA extraction from bacteria**
After incubation, the bacterial culture from each well was centrifuged at 12,000 × g for 2 min at 4 °C. The supernatant was removed, and total bacterial RNA was extracted from the pellet. The pellet was first treated with a lysis solution containing lysostaphin (0.1 mg/mL) and lysozyme (15 mg/mL) for 15–30 min at room temperature, with vortexing every 5 min. RNA was then purified using a commercial Cell Total RNA Isolation Kit (FOREGENE, China) according to the manufacturer’s instructions.

**11.3 Reverse transcription and quantitative PCR (qPCR)**
cDNA was synthesized from 2 μg of total RNA using the 5× All In One RT MasterMix (abm, USA). Quantitative PCR was performed on a QuantStudio 7 Pro Real Time PCR System (Applied Biosystems, USA) using BlasTaq™ 2× qPCR MasterMix (abm, USA). The reaction volume was 20 μL, containing 2 μL of cDNA template. The thermal cycling conditions were as follows: 95 °C for 3 min; followed by 40 cycles of 95 °C for 15 s and 60 °C for 1 min. All primer sequences are listed in Table S2. Relative gene expression levels were calculated using the 2^−ΔΔCt^ method, with the *gyrB* gene as the endogenous control.

**12. Detection of *δ*-toxin in bacterial culture supernatants**

*δ*-toxin amounts were measured by reversed-phase high pressure liquid chromatography/ electrospray mass spectrometry (RP-HPLC/ESI-MS) using an ExionLC system connected to an X500R QTOF mass spectrometer (Sciex). Samples (5 uL) were injected onto a Kinetex 2.6um C18 100Å 100×2.1mm (Phenomenex) and a 0.1% formic acid water / acetonitrile gradient was applied as follows (flow rate, 0.4 ml/min); 10% acetonitrile from 0 to 2 min, 50% acetonitrile from 2 to 4 min, 50 to 100% acetonitrile linear gradient from 4 to 9 min, 100% acetonitrile from 9 to 13 min, 10% acetonitrile from 13 to 16 min.

Relative δ-toxin production was determined by measuring the total ionic current intensity of the extracted ion chromatogram corresponding to multiply-charged m/z values of δ-toxin by using SCIEX OS software.

13. **Biofilm formation assay**

To evaluate the effect of QSI‑A on biofilm formation, an *in vitro* crystal violet staining assay was performed. *S. aureus* LAC was cultured overnight in TSB at 37 °C with shaking, then diluted 1:100 in fresh TSB containing 5% glucose. Aliquots (100 μL) of the bacterial suspension were added to 96‑well polystyrene plates and incubated with QSI‑A at concentrations corresponding to 1/8 MIC (0.3125 μmol/L), 1/4 MIC (0.625 μmol/L), and 1/2 MIC (1.25 μmol/L) of QSI‑A against LAC (MIC = 2.5 μmol/L). Untreated bacteria served as the negative control. Plates were incubated statically at 37 °C for 24 h to allow biofilm formation. After incubation, the culture medium was gently removed, and the wells were washed three times with PBS to remove non‑adherent bacteria. The adherent biofilms were fixed with methanol for 15 min, air‑dried, and then stained with 0.5% (w/v) crystal violet solution for 15 min at room temperature. Excess stain was removed by washing three times with distilled water. The crystal violet dye bound to the biofilm was solubilized with 200 μL of 33% (v/v) glacial acetic acid per well, and the absorbance was measured at 570 nm using a microplate reader. All experiments were performed in triplicate with at least three independent biological replicates.

14. **Intracellular *S. aureus* killing assay^[2]^**

The intracellular activity of QSI-A was assessed in macrophages and non-phagocytic cells. Logarithmic-phase *S. aureus* (1×10^6^ CFU/mL) was added to cell monolayers (200 μL per well in 96-well plates, containing 2×10^5^ CFU per well) for 1 h to establish infection. For RAW264.7 macrophages (seeded at 5×10^4^ cells per well), this corresponds to an MOI of 4; for A549, HaCaT, and MG63 cells (seeded at 2×104 cells per well), the same inoculum corresponds to an MOI of 10. Extracellular bacteria were removed by washing, followed by gentamicin (0.5 mg/mL, 30 min). Cells were then washed and treated with QSI-A, vancomycin, or medium alone for 4 h. After lysis with 1% Triton X-100, lysates were diluted, plated on TSA, and incubated overnight. Intracellular bacteria were quantified by CFU counting.

15. **Intracellular antibiotic release assay**

To directly verify the intracellular release of active rifalogue from QSI‑A, RAW264.7 macrophages were seeded in 96‑well plates at 5×10^4^ cells per well and allowed to adhere overnight. The cells were then incubated with QSI‑A at concentrations of 2 μmol/L for 1 h at 37 °C. After incubation, the culture medium was removed, and the cells were gently washed twice with PBS to eliminate any extracellular QSI‑A. The cells were then lysed with 0.1% Triton X‑100 (200 μL per well) to release intracellular contents. The resulting lysates were collected, serially diluted, and mixed with an equal volume of freshly prepared *S. aureus* LAC suspension (final bacterial concentration adjusted to ~1×10^6^ CFU/mL). A control group without QSI‑A treatment was included in parallel. After co‑incubation at 37 °C for 18 h, bacterial growth was assessed by visual inspection of turbidity. The MIC was defined as the lowest concentration of the cell lysate that completely inhibited visible bacterial growth.

16. **Intercellular dissemination assay**

C57BL/6J mice were infected intraperitoneally with *S. aureus* (1×10^8^ CFU). After 24 h, peritoneal lavage was performed with ice-cold PBS. Lavage fluid was centrifuged (1,000×g, 5 min, 4 °C), and the cell pellet was treated with lysostaphin (50 μg/mL, 30 min) to eliminate all extracellular and surface‑adherent bacteria, ensuring that only bona fide intracellular bacteria within macrophages were carried forward. After two washes, peritoneal macrophages were co‑cultured with MG63 osteosarcoma cells (1:1 ratio) in 96‑well plates. After 24 h, the non‑adherent macrophages were removed by gentle washing, and only the adherent MG63 cells were lysed with 0.1% Triton X‑100. Because no extracellular bacteria were present and macrophages were not lysed, the recovered CFU exclusively represent bacteria that had disseminated from macrophages into MG63 cells. The co-culture was treated with QSI-A, rifalogue, vancomycin, or PBS (control). After 24 h, MG63 cells were lysed with 0.1% Triton X-100, and intracellular bacteria were quantified by colony-forming unit (CFU) enumeration on TSA.

**17. Mouse bacteraemia infection model ^[3]^**

Female BALB/c mice (6 weeks old) were intravenously inoculated with *S. aureus* (1 × 10^6^ CFU/mouse). Twenty-four hours post-infection, mice received a single intraperitoneal dose of QSI-A (1 mg/kg, n = 3), rifalogue (0.5 mg/kg, n = 3), vancomycin (110 mg/kg, n = 3), or a physical mixture of *t*AIP‑III D4A amide and rifalogue (at equivalent doses, n = 3), or PBS (control, n = 3). After the final dose, blood, liver, kidney, and lung tissues were collected, homogenized, serially diluted, and plated on TSA. Countable colonies were recovered only from kidney homogenates; liver, lung and blood samples yielded no detectable CFU at the experimental endpoint. Bacterial loads (CFU/g tissue or CFU/mL blood) were compared between treated and control groups to assess efficacy. All animal procedures were approved by the ethics committee of the Fourth Military Medical University (Approval no. KY20212479‑C‑1).

**18. Flow cytometry binding assay ^[4]^**

**18.1 Bacterial binding assay**

Mid-log phase cultures of *S. aureus* (or *Escherichia coli* ATCC 25922) were harvested, washed, and resuspended in phosphate-buffered saline (PBS). Bacterial suspensions were then incubated with the FITC-conjugated targeting peptide at a final concentration of 5 μmol/L for 1 h at 37 °C in the dark. Following incubation, bacteria were washed twice with PBS to remove unbound peptide and resuspended in PBS for analysis. Bacteria incubated with PBS only under identical conditions served as the unstained control.

**18.2 Cellular binding assay in RAW264.7 macrophages**

RAW264.7 murine macrophages were harvested, washed, and resuspended in PBS. Cells were incubated with the FITC-conjugated targeting peptide at final concentrations of 0.5 or 1 μmol/L for 1 h at 37 °C in the dark. After incubation, cells were washed twice with PBS and resuspended in PBS for analysis. Cells treated with PBS alone served as the unstained control.

**18.3 Flow cytometry analysis**

All samples were analyzed immediately using a Beckman Coulter CytoFLEX flow cytometer. Green fluorescence was detected with excitation at 488 nm and emission collected at 533 ± 30 nm. Peptide binding was quantified as the mean fluorescence intensity (MFI), and data were processed using CytExpert software (Beckman Coulter).

**19. Confocal live-cell imaging**

Sample preparation for confocal microscopy followed the same protocol as described for flow cytometry.

Live-cell images were acquired using a ZIRCON SC3000 confocal microscope equipped with a climate chamber. Throughout the imaging process, temperature was maintained at 37 °C using a stage-top incubator. FITC fluorescence was excited with a 488 nm laser, and emission signals were collected between 500-550 nm. Bright-field or phase-contrast images were captured simultaneously to visualize cellular morphology and positioning. Scale bars represent 20 µm in all presented images.

**20. Statistical analysis**

Data are presented as mean ± SD. Statistical analyses were performed using GraphPad Prism version 9.5. For comparisons between two independent groups, an unpaired two-tailed Student's *t*-test was used. For comparisons among multiple groups against a single control, one-way ANOVA followed by Dunnett's multiple-comparisons test was used. A *P* value < 0.05 was considered statistically significant.

**Supporting figures and tables.**

**Figure S1.** Analytical LCMS chromatogram of the chlorobenzyl intermediate **4**.

**Figure S2.** Analytical LCMS chromatogram of the target product QSI-A.

**Figure S3.** Analytical HPLC chromatogram of the target product QSI-A.

**Figure S4.** QSI-A inhibits the agr QS system and associated virulence factors in MW2. **(A-E)** Relative mRNA expression levels of *agrA* **(A)**, *RNAIII* **(B)**, *psmα* **(E)**, *hla* **(D)**, and *pvl* **(E)** in *S.aureus* LAC following treatment with QSI-A at concentrations of 0.001, 0.01, 0.1, or 1 μmol/L, relative to untreated controls. *t*AIP-III D4A amide (1 μmol/L) was included as a reference agr inhibitor. **(F)** Hemolytic activity of *S.aureus* MW2 culture supernatants following QSI-A treatment (0.001, 0.01, 0.1, or 1 μmol/L). Data are presented as mean ± SD of at least three independent experiments. Statistical significance was determined by one-way ANOVA followed by Dunnett’s post-hoc test (**, *P* < 0.01; ***, *P* < 0.001; ****, *P* < 0.0001; ns, not significant), comparing all groups to the untreated infected control (or as otherwise indicated).

**Figure S5.** The influence of QSI-A on LAC biofilm production. (**A**) Bacterial OD_600_ values after 24 h culture at sub‑MIC concentrations. Blank control: sterile medium; Negative control: bacterial culture without QSI-A.(**B**) Quantitative analysis of biofilm formation using crystal‑violet assay. ns, not significant; *, p<0.05.(**C**) Visual images of crystal‑violet stained biofilms in 96‑well microplates.

**Figure S6.** QSI-A suppresses the expression of *agrA* and *RNAIII* in intracellular *S. aureus* LAC. RAW264.7 cells infected with *S. aureus* LAC were treated with the indicated concentrations of QSI A for 5 h. Total RNA was extracted from intracellular bacteria, and the relative mRNA levels of **(A)** *agrA* and **(B)** *RNAIII* were determined by RT qPCR and normalized to the housekeeping gene gyrB. Data are presented as mean ± SD of at least three independent experiments. Statistical significance was determined by one-way ANOVA followed by Dunnett’s post-hoc test (**, *P* < 0.01; ***, *P* < 0.001; ****, *P* < 0.0001; ns, not significant), comparing all groups to the untreated infected control (or as otherwise indicated).

**Figure S7.** QSI-A eliminates intracellular *S. aureus* MW2 **(A)** and N315 **(B)** in RAW264.7 cells. RAW264.7 cells were infected with *S. aureus* MW2 or N315 at an MOI of 4 and then treated with QSI A (0.5 or 1 μmol/L), vancomycin (0.1 mg/mL), or left untreated (infected control) for 4 h. Intracellular bacterial loads were determined by CFU assay. Data are presented as mean ± SD (n = 8). Statistical significance was analyzed by one way ANOVA followed by Dunnett’s post hoc test, comparing all groups to the untreated infected control: **, *P* < 0.01; ***, *P* < 0.001; ****, *P* < 0.0001; ns, not significant.

**Table S1.** Minimum Inhibitory Concentration (MIC) of antibiotics in S.aureus LAC

| Antibiotics | Minimum Inhibitory Concentration (MIC) |
| --- | --- |
| Vancomycin | 0.5 μg/mL |
| Tetracycline | 0.5 μg/mL |
| Imipenem | 0.5 μg/mL |
| Cefoperazone | 4 μg/mL |
| Rifampicin | 0.5 μg/mL |
| Rifalogue  QSI-A | 0.03125 μmol/L  2.5 μmol/L |

**Table S2.** The sequences of primers used in this study

| Primer | Sequences (5’-3’) |
| --- | --- |
| *gyrB*-forward | AGAGGTTTGCACCATTTAG |
| *gyrB*-reverse | TCCGTTATCCGTTACTTTA |
| *agrA*-forward | TGATAATCCTTATGAGGTGCTT |
| *agrA*-reverse | CACTGTGACTCGTAACGAAAA |
| *RNAIII*-forward | AATACATAGCACTGAGTCCAAGG |
| *RNAIII*-reverse | TGGATTATCGACACAGTGAACA |
| *psmα*-forward | TATCAAAAGCTTAATCGAACAATTC |
| *psmα*-reverse | CCCCTTCAAATAAGATGTTCATATC |
| *hla*-forward | GCGAAGTCTGGTGAAA |
| *hla*-reverse | GCGAAGAAGGTGCTAA |
| *pvl*-forward | CCAATAAATTCTGGATTGAAGTTACCT |
| *pvl*-reverse | GCTCAAGACAAAGCAACTTAAATGC |

**Table S3.** MIC of cell lysates from QSI-A treated macrophages against *S. aureus* LAC

| Cell lysates | MIC (%) |
| --- | --- |
| Control | >50 |
| QSI-A (2μmol/L) | 25 |

**References**

[1] Sonker, H.; Dewan, S.; Chaudhary, K.; Agrahari, B.; Rathore, N.; Jain, P.; Gautam, R. Water-Soluble Ruthenium(II) p-Cymene Complexes Mitigating Intracellular S. aureus Infection: Bridging Antibacterial Défense and Anticancer Therapy. Journal of Medicinal Chemistry 2025, 68 (16), 17897-17916.

[2] Qin, L.; Hu, N.; Zhang, Y.; Yang, J.; Zhao, L.; Zhang, X.; Yang, Y.; Zhang, J.; Zou, Y.; Wei, K. Antibody-Antibiotic Conjugate Targeted Therapy for Orthopedic Implant-Associated Intracellular S. aureus Infections. Journal of Advanced Research 2024, 65 (2024), 239-255.

[3] M., S.; Pillow, T.; Xu, M.; Staben, L.; K., K.; Vandlen, R.; DePalatis, L.; Raab, H.; L., W.; Hiroshi, J. Novel Antibody-Antibiotic Conjugate Eliminates Intracellular S. Aureus. Nature 2015, 527 (7578), 323-328.

[4] Tsuchikama, K.; Shimamoto, Y.; Anami, Y. Truncated Autoinducing Peptide Conjugates Selectively Recognize and Kill Staphylococcus Aureus. ACS Infectious Diseases 2017, 3 (6), 406-410.
